# Mouse Model of Congenital Heart Defects, Dysmorphic Facial Features and Intellectual Developmental Disorders as a Result of Non-functional CDK13

**DOI:** 10.3389/fcell.2019.00155

**Published:** 2019-08-07

**Authors:** Monika Nováková, Marek Hampl, Dávid Vrábel, Jan Procházka, Silvia Petrezselyová, Michaela Procházková, Radislav Sedláček, Michaela Kavková, Tomáš Zikmund, Jozef Kaiser, Hsien-Chia Juan, Ming-Ji Fann, Marcela Buchtová, Jiří Kohoutek

**Affiliations:** ^1^Department of Chemistry and Toxicology, Veterinary Research Institute, Brno, Czechia; ^2^Laboratory of Molecular Morphogenesis, Institute of Animal Physiology and Genetics, Czech Academy of Sciences, Brno, Czechia; ^3^Department of Experimental Biology, Faculty of Science, Masaryk University, Brno, Czechia; ^4^Laboratory of Transgenic Models of Diseases, Institute of Molecular Genetics, Czech Academy of Sciences, Prague, Czechia; ^5^Czech Centre for Phenogenomics, Institute of Molecular Genetics, Czech Academy of Sciences, Prague, Czechia; ^6^Central European Institute of Technology, Brno University of Technology, Brno, Czechia; ^7^Department of Life Sciences, Institute of Genome Sciences, National Yang-Ming University, Taipei, Taiwan

**Keywords:** cyclin-dependent kinase (CDK), cyclin, transcription regulation, development, mouse, cyclin-dependent kinase 13, cyclin K

## Abstract

Congenital heart defects, dysmorphic facial features and intellectual developmental disorders (CHDFIDD) syndrome in humans was recently associated with mutation in *CDK13* gene. In order to assess the loss of function of Cdk13 during mouse development, we employed gene trap knock-out (KO) allele in *Cdk13* gene. Embryonic lethality of Cdk13-deficient animals was observed by the embryonic day (E) 16.5, while live embryos were observed on E15.5. At this stage, improper development of multiple organs has been documented, partly resembling defects observed in patients with mutated CDK13. In particular, overall developmental delay, incomplete secondary palate formation with variability in severity among Cdk13-deficient animals or complete midline deficiency, kidney failure accompanied by congenital heart defects were detected. Based on further analyses, the lethality at this stage is a result of heart failure most likely due to multiple heart defects followed by insufficient blood circulation resulting in multiple organs dysfunctions. Thus, Cdk13 KO mice might be a very useful model for further studies focused on delineating signaling circuits and molecular mechanisms underlying CHDFIDD caused by mutation in *CDK13* gene.

## Introduction

Recently, *de novo* missense variants in *Cyclin-dependent kinase 13* (*CDK13*) gene have been identified as an emerging factor involved in the onset of congenial heart defects (CHD) in humans (Sifrim et al., 2016). Documented CHD cases were characterized by ventral and atrial septal defects accompanied by pulmonary valve abnormalities. CHD patients had syndromic facial gestalt, and two patients had agenesis of the corpus callosum. Additional mutations within the *CDK13* gene were recognized in humans resembling at clinical level many symptoms previously associated with loss of function of *CDK13* or newly described symptoms, such as autism spectrum disorder, seizures, feeding difficulties and craniofacial dysmorphism including short upslanting palpebral fissures, hypertelorism or telecanthus, medial epicathic folds, low-set, posteriorly rotated ears and small mouth with thin upper lip vermilion (Hamilton et al., 2018). In addition, genome wide search for *de novo* mutations responsible for developmental disorders in patients in Great Britain and Republic of Ireland led to identification of 14 genes that previously lacked compelling evidence of involvement in developmental disorders, among them the *CDK13* gene (Deciphering Developmental Disorders Study, 2017). However, heart defects do not seem to be the key feature of this disorder since patients with heterozygous constitutional mutation in *CDK13* lacking cardiac anomalies were reported by two groups (Carneiro et al., 2018; Uehara et al., 2018). Spectrum of clinical phenotypes of patients with *CDK13* mutations varies from mild to severe with the ubiquitous intellectual disability and developmental delay (ID/DD) (van den Akker et al., 2018). Based on these observations, congenital heart defects, dysmorphic facial features and intellectual development disorder (CHDFIDD) have been recognized as novel syndrome caused by *de novo* variants of *CDK13* gene (Sifrim et al., 2016; Bostwick et al., 2017; Carneiro et al., 2018; van den Akker et al., 2018).

Human CDK13 protein consists of 1512-amino acids with a conserved kinase domain surrounded by N- and C-terminal arms of undefined function (Kohoutek and Blazek, 2012). In order to be active, CDK13 binds cyclin K (CycK) and forms enzymatically active complex (Ko et al., 2001; Even et al., 2006; Bartkowiak et al., 2010; Blazek et al., 2011; Cheng et al., 2012; Dai et al., 2012; Kohoutek and Blazek, 2012; Liang et al., 2015). CDK13 belongs to the family of transcription-associated cyclin-dependent kinases phosphorylating the carboxyl-terminal domain (CTD) of RNA polymerase II (RNAPII). In particular, the CDK13 phosphorylates serine 2 (Ser2) and to a lesser extend also serine 5 (Ser5) within Y^1^S^2^P^3^T^4^S^5^P^6^S^7^ heptapeptides within the CTD of RNAPII *in vitro* (Greifenberg et al., 2016). Nevertheless, downregulation of CDK13 in tumor derived cell lines had a very small, if any, effect on level of Ser2 within CTD of RNAPII (Blazek et al., 2011; Greifenberg et al., 2016). In parallel to CDK13, there is CDK12 in mammalian cells able to associate with CycK as well (Blazek et al., 2011; Dai et al., 2012; Liang et al., 2015). Even though CDK13 shares high amino acid similarity with CDK12, both kinases appear to function in mutually exclusive complexes in mammalian cells (Blazek et al., 2011; Kohoutek and Blazek, 2012; Greenleaf, 2018).

In comparison to CDK12, there is a limited number of papers envisioning the likely function of CDK13 in various biological processes. For instance, the CDK13 was proposed to be involved in oncogenesis; yet, its precise function is still under examination (Kim et al., 2012; Pan et al., 2012). Even though factors involved in RNA processing, RNA splicing, polyadenylation and RNA cleavage were demonstrated to bind CDK13 as a result of global protein-protein interactions, truly associating partners of this kinase are still unknown (Davidson et al., 2014; Bartkowiak and Greenleaf, 2015; Liang et al., 2015). In addition to involvement of CDK13 in diverse cellular processes, this protein participates in regulation of alternative splicing of HIV-1 or influenza virus replication, thus suppressing viral production (Berro et al., 2008; Bakre et al., 2013). In developing mouse embryos and murine cells, CDK13 regulates hematopoiesis, stemness and axonal elongation, suggesting an important function in neuronal development (Pan et al., 2012; Chen et al., 2014).

To this date, the impact of complete loss of *Cdk13* function during mammalian development has not been investigated. Therefore, we employed a *Cdk13* knock-out (KO) mouse model to explore a novel role of *Cdk13* during mouse embryonic development. We observed embryonic lethality of *Cdk13* KO animals at the embryonic day 16.5. At this stage, improper development of multiple organs has been observed (heart, brain, kidney, liver, and palate formation) resembling phenotype of human patients with *de novo* missense variants of *CDK13* gene. Therefore, our *Cdk13*-deficient mice may become an important model to study dysregulation of developmental processes occurring in human patients.

## Results

### Disruption of Cdk13 Gene Leads to Embryonic Lethality in Mice

**FIGURE 1:**
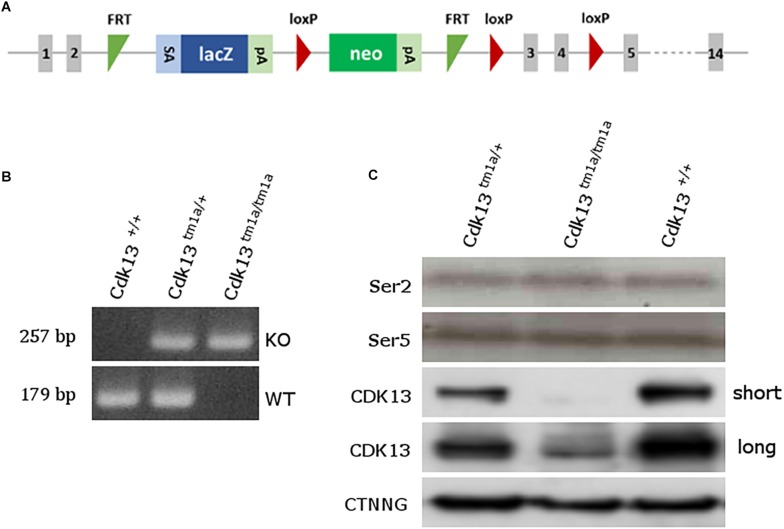
Generation of *Cdk13^*tm*1*a*^* mice. **(A)** Scheme of *Cdk13^*tm*1*a*^* allele. The *Cdk13^*tm*1*a*^* allele consists of strong splicing acceptor (SA), β-galactosidase gene (lacZ) and neomycine resistance gene (neo) both with poly A sites (pA), being surrounded by two FRT sites within intron 2, having two and one lox P sites within intron 2 and 4. **(B)** PCR genotyping from E12.5 embryos using specific primers distinguishing *Cdk13^*tm*1*a*^*, mutant (KO, upper bands) and *Cdk13*^+^, wild-type, allele (WT, lower bands). **(C)** Brain extracts were prepared from E14.5 embryos of *Cdk13*^+/+^, *Cdk13^*tm*1*a/+*^* and *Cdk13^*tm*1*a/tm*1*a*^* mice and protein levels of Ser2 and Ser5 of RNAPII, CDK13 and γ-catenin (CTNNG) were evaluated by Western blotting. The short and long expositions for CDK13 are presented to demonstrate residual expression of CDK13 in *Cdk13^*tm*1*a/tm*1*a*^* embryos.

To examine the role of CDK13 during mouse development, the mice carrying *Cdk13^*tm*1*a*^* allele were generated at the Transgenic and Archiving Module CCP (Institute of Molecular Genetics of the CAS, Prague). The *Cdk13^*tm*1*a*^* allele of *Cdk13* gene enables cessation of transcription due to presence of two strong poly A sites leading to production of the aberrant transcript of *Cdk13* mRNA resulting in non-functional truncated form of CDK13 protein harboring only N-terminal part of this protein, without kinase domain and C-terminal part (Figure 1A). Heterozygous *Cdk13^*tm*1*a/+*^* mice were intercrossed to obtain *Cdk13^*tm*1*a/tm*1*a*^* offspring. Newborn mice were genotyped with specific sets of primers able to distinguish inserted cassette (257 bp PCR product, KO) and wild-type (179 bp PCR product, WT) alleles of *Cdk13* gene (Figure 1B). Although the offspring with *Cdk13* WT and heterozygous alleles was born at the expected Mendelian ratio, appeared normal and fertile, *Cdk13^*tm*1*a/tm*1*a*^* mice were not born at all. This finding suggested that a homozygous deficiency in *Cdk13* gene leads to embryonic lethality in mice. To determine the precise stage, when the embryonic lethality occurs, mouse embryos were collected at various gestation time points (Table 1). There were no living *Cdk13^*tm*1*a/tm*1*a*^* embryos after the embryonic stage 15.5 (E15.5) judging by the lack of their heart beating. Moreover, from E13.5 to E16.5, we observed increased number of absorbed embryos as a reflection of empty decidua (Table 1). Based on these finding, we concluded that the deficiency of *Cdk13* causes severe adverse developmental defects and consequent death from E14.5 resulting in total lethality before E16.5.

**TABLE 1:** Genotypes of offspring from *Cdk13*^*tm1a/+*^ intercross.

					**Empty**
**Stage**	***Cdk13*^+/+^**	***Cdk13*^*tm1a/+*^**	***Cdk13*^*tm1a/tm1a*^**	**Litters**	**decidua**
	**(25%)**	**(50%)**	**(25%)**		
E9.5	4 (21.1%)	12 (63.2%)	3 (15.8%)^*^	3	2
E10.5	4 (20%)	10 (50%)	6 (30%)^*^	2	0
E11.5	14 (31.8%)	23 (52.3%)	7 (15.9%)^*^	6	4
E12.5	31 (30.7%)	52 (51.5%)	18 (17.8%)^*,#^	15	13
E13.5	6 (16.2%)	18 (48.6%)	13 (35.1%)^*^	5	5
E14.5	48 (27.6%)	95 (54.6%)	31 (17.8%)^*,#^	26	9
E15.5	21 (27.5%)	34 (58%)	10 (14.5%)^*^	11	5
E16.5	8 (25%)	18 (56.3%)	6 (18.7%)^*,#^	4	0
P0	39 (33.1%)	79 (66.9%)	0	28	

^*^Growth retardation, #dead embryo.

To confirm the loss of CDK13 protein in *Cdk13^*tm*1*a/tm*1*a*^* mice, developing brain from WT and *Cdk13^*tm*1*a*/tm1*a*^* homozygous embryos at E14.5 were collected and western blot analyses were carried out with specific antibodies recognizing CDK13 protein. As expected, the WT form of CDK13 was present in *Cdk13*^+/+^ and *Cdk13^*tm*1*a/+*^* mice, but surprisingly, corroborated residual expression of CDK13 protein was detected in the embryonic brain of *Cdk13^*tm*1*a*/tm1*a*^* homozygous embryos (Figure 1C), suggesting that *Cdk13^*tm*1*a/tm*1*a*^* mice represent a hypomorphic mutant phenotype. Because the anti-Cdk13 antibody used in western blot recognizes its N-terminal part of CDK13, we were curious if the truncated form of CDK13, as a result of terminated transcription within intron 2, will be expressed in mice bearing the *Cdk13^*tm*1*a*^* allele. Indeed, the truncated form of CDK13 was detected in *Cdk13^*tm*1*a/+*^* and *Cdk13^*tm*1*a/tm*1*a*^* animals (Supplementary Figure S1). Since CDK13 was demonstrated to phosphorylate CTD of RNAPII *in vitro*, we decided to evaluate phosphorylation status of CTD in hypomorphic mice. Thus, the effect of CDK13 downregulation on Ser2 was evaluated in the animal tissue. Even though expression of CDK13 was significantly lowered in *Cdk13^*tm*1*a/tm*1*a*^* mice, no effect on either Ser2 or Ser5 phosphorylation within CTD of RNAPII was detected (Figure 1C). The Ser5 phosphorylation was checked in parallel since it is phosphorylated by other CDK kinase, CDK7 in particular (Kohoutek, 2009).

### Cdk13 Loss Causes Growth Retardation, Developmental Delay and Its Failure

**FIGURE 2:**
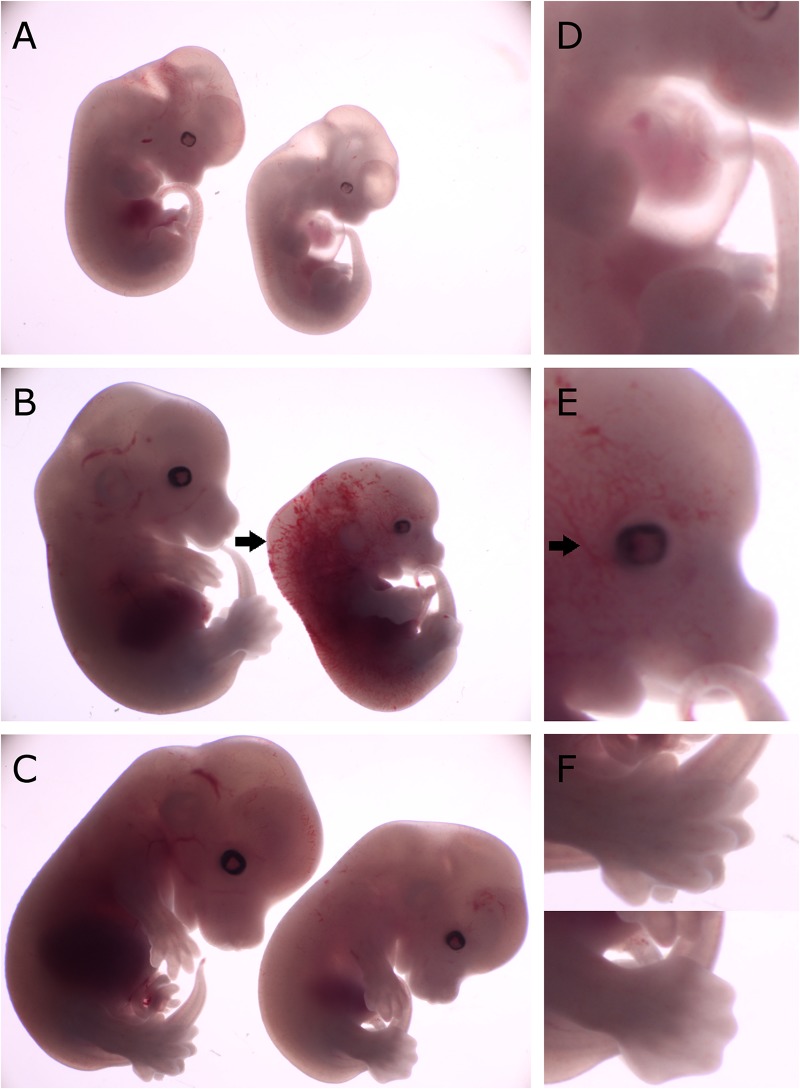
Comparison of gross morphology of wild-type, *Cdk13^*tm*1*a/+*^* and *Cdk13^*tm*1*a/tm*1*a*^* embryos at various stages. *Cdk13^*tm*1*a/tm*1*a*^* embryos display significant growth retardation compared to wild-type and heterozygous embryos. **(D–F)** Detailed images of *Cdk13^*tm*1*a/tm*1*a*^* at relevant developmental stages. **(A)** Heterozygous and *Cdk13^*tm*1*a/tm*1*a*^* embryos at E12.5. **(D)** Occasional chest wall deformities manifest at hypomorphs. **(B)** Wild-type and *Cdk13^*tm*1*a/tm*1*a*^* embryos at E13.5. *Cdk13^*tm*1*a/tm*1*a*^* embryo exhibits nuchal edema (black arrow). **(E)** Hypervascularization of the peripheral vessels capillaries (black arrow). **(C)** Wild-type and *Cdk13^*tm*1*a/tm*1*a*^* embryos at E14.5. (**F**, top) Wild-type embryo demonstrates deep indentations between the developing fingers of embryos E14.5, although not yet separated. (**F**, bottom) In contrast, *Cdk13^*tm*1*a/tm*1*a*^* embryo appears to be 1 day delayed in development as evidenced by the shallow indentation of the footpad, which is characteristic of embryos E13.5.

To examine deficiency of CDK13 protein in *Cdk13^*tm*1*a/tm*1*a*^* mice, morphology at different embryonic stages compared to the WT animals was examined and various abnormalities were observed in *Cdk13^*tm*1*a/tm*1*a*^* embryos at each stage of gestation (Supplementary Figure S2). Observed growth retardation of *Cdk13*-deficient mice is presented in detail (Figure 2); however, the severity of the developmental delay was variable. On average, the *Cdk13^*tm*1*a/tm*1*a*^* embryos appeared to be one embryonic day behind in comparison to their littermate controls as evidenced by the shallow indentation of the footpad, which is characteristic of embryos at E13.5 (Figure 2F, bottom). In contrast, *Cdk13*^+/+^ littermates exhibited deep indentations between the developing toes (not yet separated), what is characteristic of embryos at 14.5 (Figure 2F, top). In addition, the retarded embryo exhibited nuchal edema (black arrow, Figure 2B), which correlates with the presence of cardiovascular phenotypes. Occasionally, the pericardial effusion were detected in developing *Cdk13^*tm*1*a/tm*1*a*^* embryos, most likely caused by dysfunction of the heart (Figure 2D). Summary of various developmental defects associated with hypomorphic *Cdk13^*tm*1*a*^* allele is presented in Supplementary Table S1.

### Cdk13 Is Indispensable for the Development of Several Organs

**FIGURE 3:**
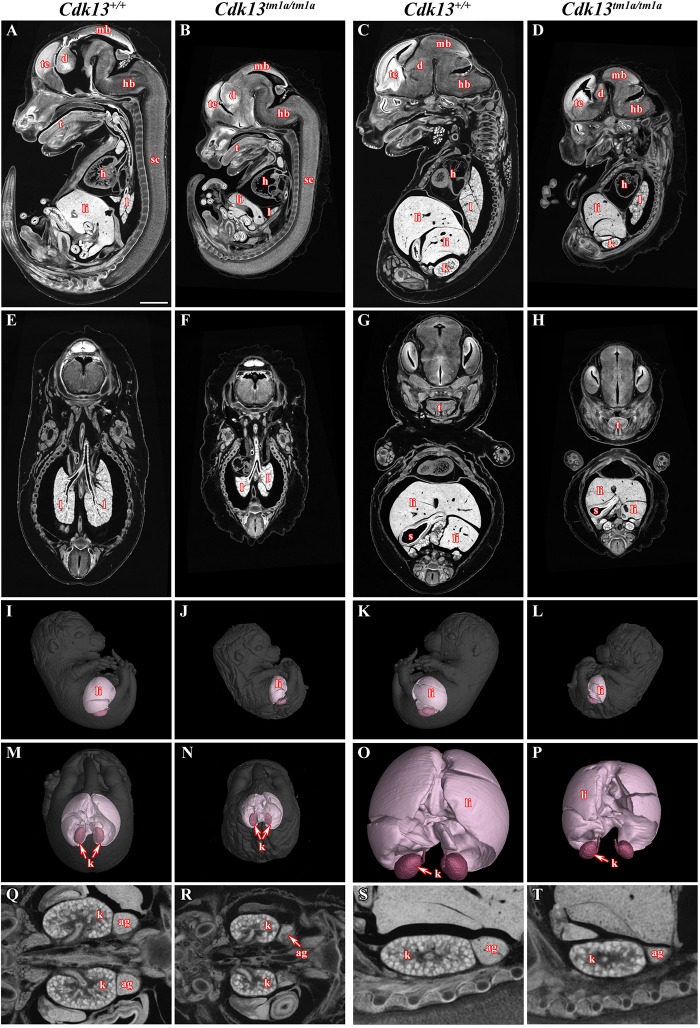
MicroCT analysis of wild-type and *Cdk13^*tm*1*a/tm*1*a*^* embryos. High-contrast differentiation resolution by X-ray computed microtomography, where Lugol’s staining was used to visualize the soft tissues. Sagittal sections through body midline **(A,B)** and more lateral plane at E15.5 **(C,D)**. Horizontal sections through lung **(E,F)** and liver **(G,H)**. 3D reconstruction of kidney and liver in the right side view of embryo **(I,J)**, left side view **(K,L)** and caudal view **(M,N)** with embryo outlined in gray where segmentation of serial sections was used for the liver and kidney reconstruction. **(O,P)** High power of 3D imaging for liver and kidney. Horizontal view **(Q,R)** and sagittal detailed view **(S,T)** on kidney and suprarenal gland. Abbreviation used for individual organs: ag, adrenal gland; d, diencephalon; h, heart; hb, hindbrain; k, kidney; l, lung; li, liver; mb, midbrain; t, tongue; te, telencephalon; s, stomach; sc, spinal cord. Scale bar = 1 mm.

To narrow down possible cause of embryonic lethality, embryos at E14.5 were contrasted with Lugol’s solution to visualize gross morphology of individual soft tissues by microCT (Figure 3). Indeed, several developmental abnormalities were detected within developing embryos. The heart wall of both ventricles in *Cdk13^*tm*1*a/tm*1*a*^* embryos (Figures 3B,D) appeared thinner in comparison to *Cdk13*^+/+^ littermate controls (Figures 3A,C). In addition, lung, liver and kidney in *Cdk13^*tm*1*a/tm*1*a*^* embryos were smaller and undeveloped (Figures 3D,F,H) in comparison to *Cdk13*^+/+^ littermates (Figures 3C,E,G). However, detailed 3D reconstruction of liver and kidney (Figures 3I–P) with movable display of E14.5 embryos of *Cdk13^*tm*1*a/tm*1*a*^* and *Cdk13*^+/+^ genotypes (Supplementary Figures S3A,B) revealed no defect in general gross morphology of these organs.

**FIGURE 4:**
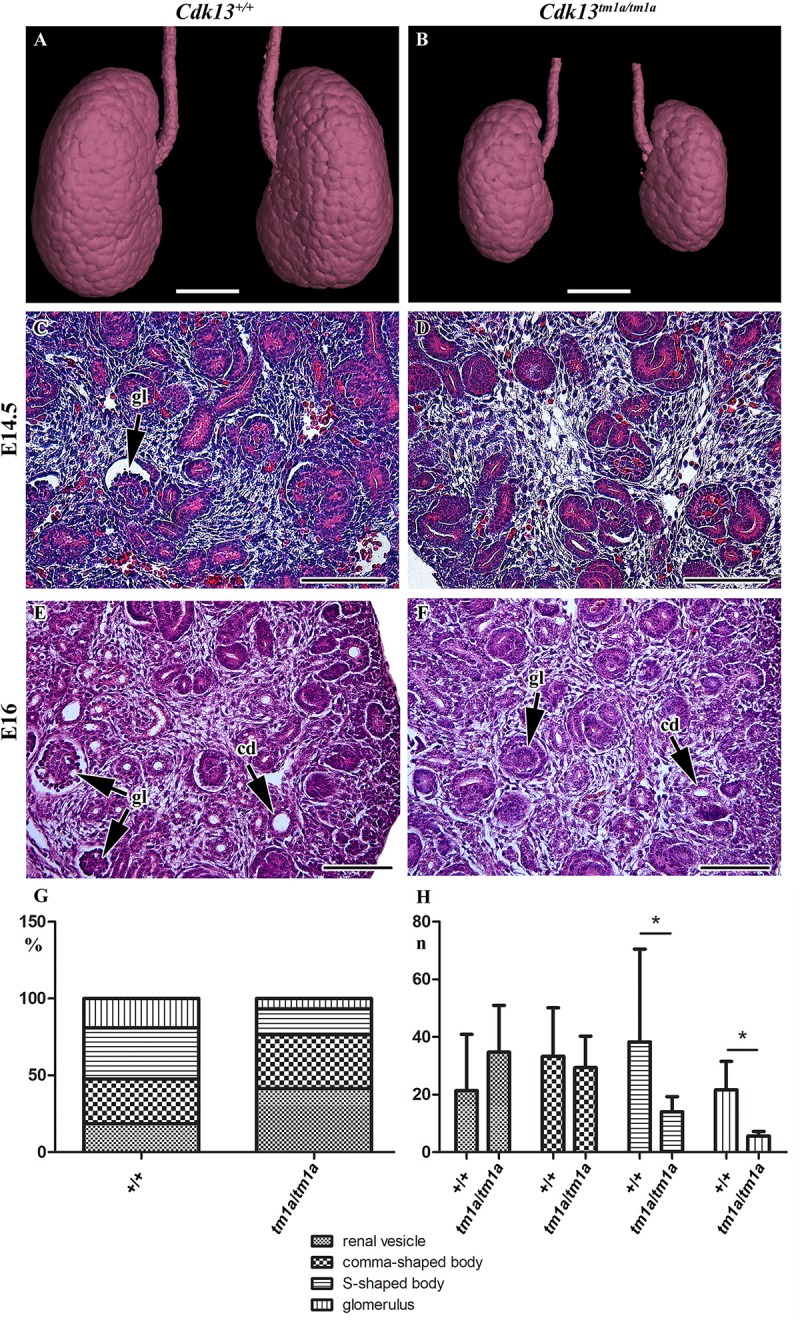
Gross anatomy and microscopic structure of kidney in wild-type and *Cdk13^*tm*1*a/tm*1*a*^* embryos. **(A,B)** High power view on segmented kidneys from Lugol’s stained sections and visualized by X-ray computed microtomography. Growth retardation of kidney is visible in *Cdk13^*tm*1*a/tm*1*a*^* embryos at E14.5 **(D)** and E16 **(F)** in contrast to littermate wild-type, *Cdk13*^+/+^, controls (**C** for E14.5 and **E** for E16). **(E,F)** Only very few just forming glomeruli (gl) were found in *Cdk13^*tm*1*a/tm*1*a*^* embryos. **(G)** Relative quantification of individual developmental stages of nephrogenesis in *Cdk13*^+/+^ and *Cdk13^*tm*1*a/tm*1*a*^* embryos. **(H)** Increased amount of renal vesicles together with the reduction of S-shaped bodies and glomeruli (gl) was found in *Cdk13^*tm*1*a/tm*1*a*^* embryos. The graph values denote median ± s.d., ^*^p < 0.05, by unpaired *t*-test. Scale bar **(A,B)** = 0.3 mm, scale bar **(C–H)** = 100 μm.

To uncover possible discrepancies in developmental speed of individual organs, the volume analysis was performed with normalization to total body volume of given embryo. Liver size of *Cdk13^*tm*1*a/tm*1*a*^* embryos represented only about 46% in comparison to *Cdk13*^+/+^ littermates (Figures 3, 11). Similarly, kidney size of *Cdk13^*tm*1*a/tm*1*a*^* animals comprised only about 52% in comparison to *Cdk13*^+/+^. In parallel, histological sections of selected organs were analyzed at stages between E14.5 and E16 (Figure 4). Decelerated development of kidneys was identified in *Cdk13^*tm*1*a/tm*1*a*^* embryos at E14.5 and E16 (Figures 4D,F) including nephron differentiation as shown by altered proportional representation of individual nephron stages at E14.5 (Figure 4G). Moreover, statistically significant reduction in the number of S-shaped bodies and glomeruli was observed in *Cdk13^*tm*1*a/tm*1*a*^* embryos (Figure 4H). At lethality stage E16.5, kidney tissue exhibited tissue abrogation with only few, much reduced tubules visible (data not shown), very likely caused by general pre-necrotic changes.

**FIGURE 5:**
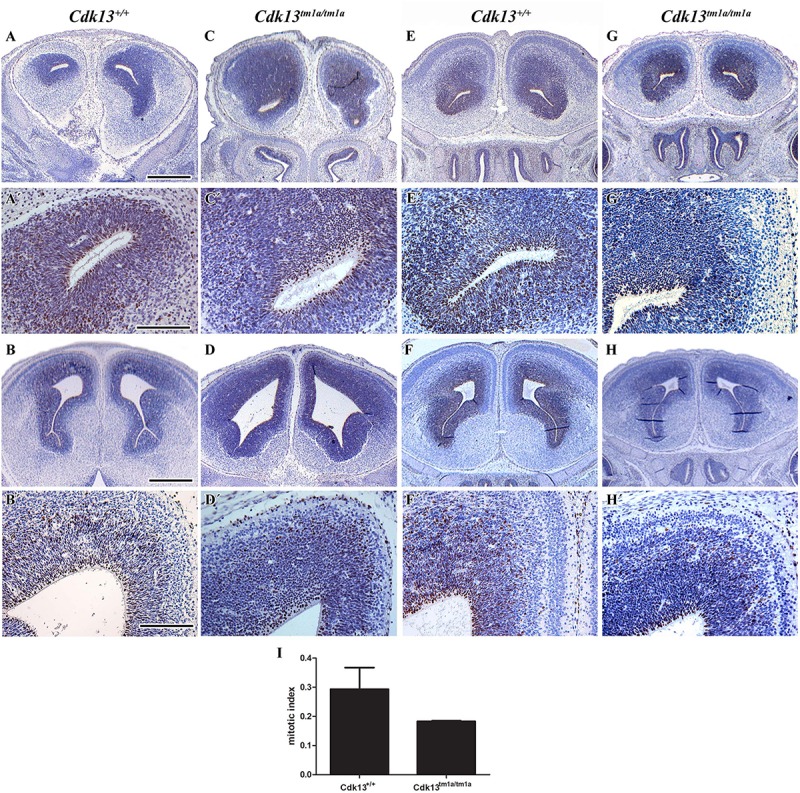
Cell proliferation in brain area in wild-type and *Cdk13^*tm*1*a/tm*1*a*^* embryos. **(A,A’,B,B’)** *Cdk13*^+/+^ and **(C,C’,D,D’)** *Cdk13^*tm*1*a/tm*1*a*^* embryos before palatal shelves fusion. **(E,E’,F,F’)** *Cdk13*^+/+^ and **(G,G’,H,H’)** *Cdk13^*tm*1*a/tm*1*a*^* embryos after palatal shelves fusion. Immunohistochemical nuclear labeling of Ki67-positive cells in the frontal head sections in the lower power view **(A–H)** and in detail **(A’–H’)**. **(I)** Mitotic index was counted as the ratio between Ki67-positive cells and total amount of prosencephalon cells in three biological triplicates for each group. The graph values denote mean ± s.d, difference is not statistically significant according to unpaired *t*-test (*p*-value: 0.1047). Ki67-positive cells - brown nuclei, Ki67-negative cells - blue nuclei (hematoxylin). Scale bar **(A–C)** = 1 mm; scale bar **(A’–C’)** = 100 μm.

Brains of Cdk13^*tm*1*a/tm*1*a*^ embryos appeared developmentally delayed as demonstrated by reduced size as compared to littermate controls. Depicted in Figure 5 are E14.5 controls and Cdk13^*tm*1*a/tm*1*a*^ mutant samples from two separate litters. The two litters were developmentally at different stages, pre-palatal fusion in the control embryo depicted (Figures 5A,B) and post-palatal fusion in the control embryo depicted (Figures 5E,F). In order to assess the developmental delay in *Cdk13^*tm*1*a/tm*1*a*^* embryos the cell proliferation was examined by Ki67 staining, a marker of proliferating cells (Figures 5A’–H’, embryos A and C, as well as E and G are littermates, representative pictures of two embryos are shown to display variability in mutant phenotype). As evident from performed quantification, there was decrease in number of proliferating cells in *Cdk13^*tm*1*a/tm*1*a*^* embryos in comparison to *Cdk13*^+/+^ littermate controls, but this decrease was not statistically significant (Figure 5I).

**FIGURE 6:**
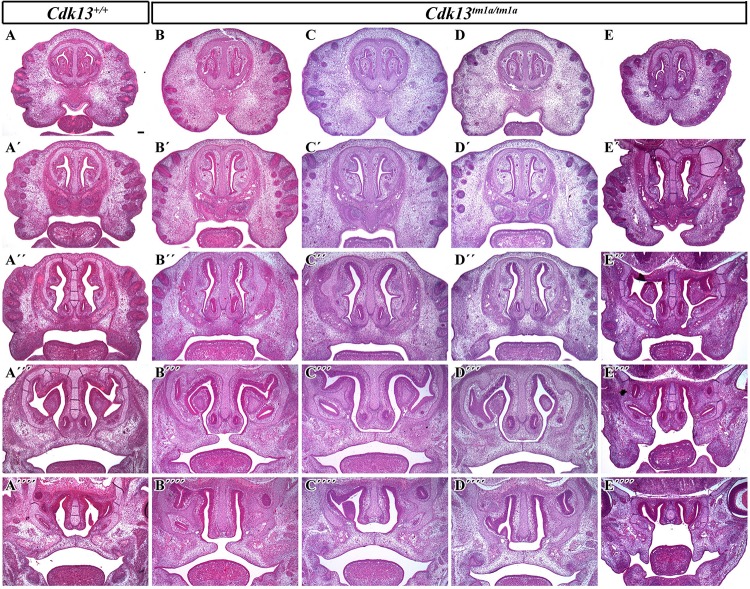
Transversal sections of the head in control *Cdk13*^+/+^ and *Cdk13^*tm*1*a/tm*1*a*^* embryos at E15.5. Rostro-caudal view in *Cdk13*^+/+^ animal **(A)** and four *Cdk13^*tm*1*a/tm*1*a*^* mutant mice to show variability in the secondary palate morphology **(B–E)**. Palatal shelves do not meet each other in the midline **(B”’,E”’)** and cleft of secondary palate is visible. Abnormal shape of palatal shelves was observed also caudally with cleft expanding into the soft palate area. **(A’–A””, B’–B””, C’–C””, D’–D””)** are transversal sections of head in individual embryos in rostrocaudal direction. Scale bar = 100 μm.

The analysis of craniofacial area revealed also defective palatal shelves development in several *Cdk13^*tm*1*a/tm*1*a*^* embryos resulting in their insufficient horizontal growth and the formation of the cleft palate at E15.5 (Figure 6) in *Cdk13^*tm*1*a/tm*1*a*^* mouse. Incomplete secondary palate formation exhibited variability in severity among *Cdk13*-deficient animals. Observed penetrance of secondary cleft palate was 2/4 animals at E15.5. In addition, *Cdk13^*tm*1*a*/tm1*a*^* mouse at E15.5 had smaller number of initiated nasal glands in comparison to the controls (Supplementary Figure S4, compare A–D and B–E).

### Embryonic Lethality in *Cdk13^*tm*1*a/tm*1*a*^* Mice Is Due to Heart Failure

**FIGURE 7:**
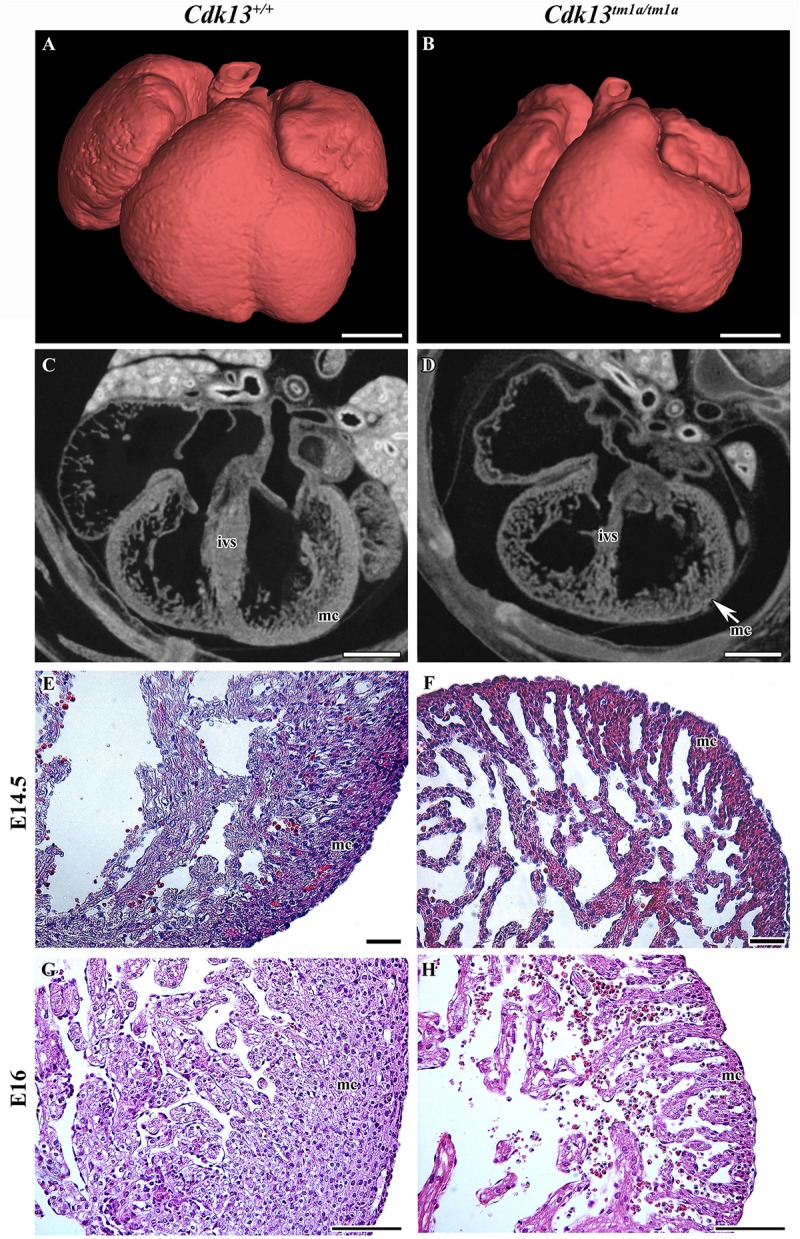
Gross anatomy and microscopic structure of heart in wild-type and *Cdk13^*tm*1*a/tm*1*a*^* embryos. **(A,B)** High power view on segmented heart from Lugol’s stained sections and visualized by X-ray computed microtomography. **(C,D)** Sagittal section of heart from Lugol’s staining visualized by X-ray computed microtomography. Smaller size of heart with broadening of ventricles was visible in *Cdk13^*tm*1*a/tm*1*a*^* animals. Abbreviations for the organ structures: ivs, interventricular septum; mc, myocardium. **(E,F)** At E14.5, myocardium (mc) of *Cdk13^*tm*1*a/tm*1*a*^* mice is already thinner in the ventricle area with small amount of cardiomyocytes layers, which is in contrast to thick wall of *Cdk13*^+/+^ littermate controls. Later, apparent disruption of tissue architecture with enlarged intercellular spaces and small amount of cardiomyocytes was observed in *Cdk13^*tm*1*a/tm*1*a*^* embryos at later stages at E16 **(G,H)**. Scale bar **(A,B)** = 0.4 mm, scale bar **(C,D)** = 350 μm, and scale bar **(E–H)** = 100 μm.

The heart is the one of the first organs to form during mammalian development. During heart development, significant changes in organ morphology and cardiomyocyte differentiation and organization reflect the increasing needs of growing embryos for nutrition and oxygen supply. Any of these developmental steps are critical for further development of whole embryos. Therefore, we analyzed the microscopic structure of the heart and found that the heart wall of *Cdk13^*tm*1*a/tm*1*a*^* mice embryos was less compact in comparison to the heart wall of *Cdk13*^+/+^ mice (Figures 7C,D). Further, apparent disruption of tissue architecture was detected in *Cdk13^*tm*1*a/tm*1*a*^* embryos with the reduction of myocardium (Figure 7, compare C, E and G to D, F and H). The heart volume was slightly increased to 103% (organ ratio to total body volume) in *Cdk13^*tm*1*a/tm*1*a*^* mice compared to *Cdk13*^+/+^ animals. However, the total volume of heart tissue to the organ volume was significantly lower in case of *Cdk13^*tm*1*a/tm*1*a*^* embryos (62.5%) in comparison to WT littermates (80%) suggestive of the thinner heart wall at E15.5 in *Cdk13^*tm*1*a/tm*1*a*^* embryos. Percent soft tissue volumes were measured by microCT using Bruker-microCT CT-analyzer, where the object volume representing soft tissues was divided by total VOI volume. The VOI area referring to the heart volume was selected from the dataset manually. Also, decreased expression of myosin was detected in ventricle myocardium of 14.5 hearts (Supplementary Figure S5, compare B and B’ to E and E’).

**FIGURE 8:**
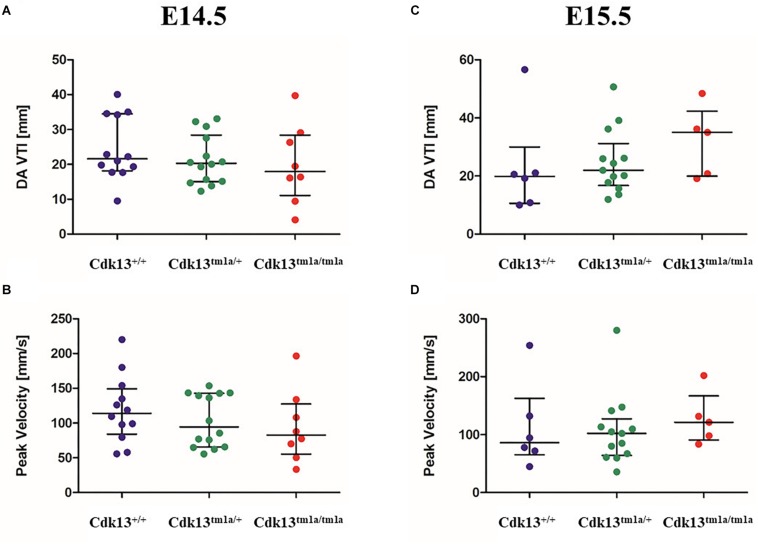
Hemodynamic analysis of wild-type, *Cdk13^*tm*1*a/+*^* and *Cdk13^*tm*1*a/tm*1*a*^* embryos. The non-invasive ultrasound Doppler imaging to quantitatively assess the heart hemodynamic function in E14.5 and E15.5 embryos was carried out. **(A,C)** Velocity-time integral in dorsal aorta (DA VTI) and **(B,D)** Peak Doppler blood flow velocity measured on E14.5 **(A,B)** and E15.5 **(C,D)** day old embryos, respectively.

Since the heart mass of *Cdk13^*tm*1*a/tm*1*a*^* E14.5 – E16 embryos is significantly reduced with hypomorphic muscular layers of myocardium compared with *Cdk13*^+/+^ mice (heart/body ratio), we presume that heart developmental defect is the cause of embryonic lethality. In order to evaluate cardiac circulatory physiology, we performed non-invasive ultrasound Doppler imaging to quantitatively assess the hemodynamic function in E14.5 and E15.5 embryos. Out of nine *Cdk13^*tm*1*a/tm*1*a*^* embryos dissected at E14.5 stage, only one was found dead (Table 2). All the other *Cdk13^*tm*1*a/tm*1*a*^* embryos at this stage exhibited comparable blood flow velocities and velocity-time integral (VTI) in dorsal aorta (Figures 8A,B). However, this situation changed dramatically at embryonic stage E15.5 (Figures 8C,D), where only few *Cdk13^*tm*1*a/tm*1*a*^* embryos retained normal blood flow parameters (5/16), while the rest of the embryos heart functions declined (standard measurements were not possible due to irregular or spare heart beating 11/16); moreover, an increased portion of embryos were already dead (Table 2).

**TABLE 2:** Summary of ultrasound scanned embryos at E14.5 and E15.5.

**Stage**	**Number of embryos**			**Litters**	**Number of *Cdk13^*tm*1*a/tm*1*a*^***	
	***Cdk13*^+/+^**	***Cdk13^*tm*1*a/+*^***	***Cdk13^*tm*1*a/tm*1*a*^***		**Heart beating**	**Dying/dead**
E14.5	11	27	9	6	8	1
E15.5	14	36	17	8	6	11

The assessment of cardiac function in developmental interval E14.5 – E15.5 embryos showed dramatic failure in heart function, probably corresponding to increasing needs of embryos at E15.5 for blood supply in growing organ systems, which is very challenging for the defective heart to achieve. The preserved blood flow in few *Cdk13^*tm*1*a/tm*1*a*^* embryos was probably due to the observed variability of the embryo size. Our findings suggest that the heart failure appears in most cases during the transition from E14.5 to E15.5.

### Cdk13 Is Expressed in Affected Organs in the Prenatal and Also Postnatal Period

**FIGURE 9:**
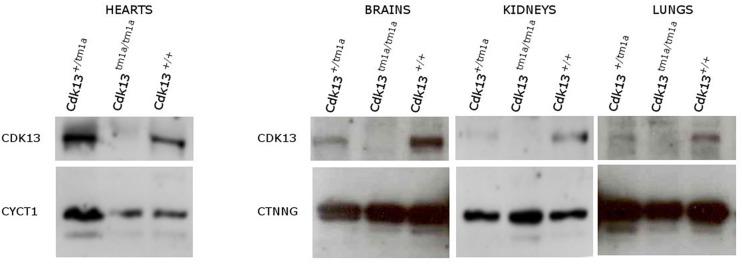
Expression of CDK13 protein in embryonic mouse organs. Protein extracts were obtained from brain, heart, kidneys and lung of E14.5 *Cdk13*, *Cdk13^*tm*1*a/+*^* and *Cdk13^*tm*1*a/tm*1*a*^* embryos and protein levels of CDK13, Cyclin T1 (CYCT1) and γ-catenin (CTNNG) were evaluated by Western blotting. The highest expression of CDK13 was observed in brain and kidney, with substantial expression in lung and heart in *Cdk13*^+/+^ embryos.

Currently, there is limited information about protein expression pattern of CDK13 either in developing or adult organs; therefore, we decided to evaluate expression of CDK13 protein in developing organs. First, the western blot of CDK13 protein was carried out in selected developing organs (Figure 9). As expected, expression of CDK13 was detected in organs with abnormal embryonic development. The highest protein level of CDK13 was detected in the brain, then lung, kidney and heart (Figure 9). In case of detection of CDK13 in the developing heart, four times concentrated protein lysates had to be used to detect any reproducible signal.

To explore gene expression of *Cdk13* in adult tissues and organs, the particular organs were isolated and the expression of Cdk13 was examined by activity of β-galactosidase (Supplementary Figure S6). *Cdk13* was strongly expressed in the retina of the eye, testes, ovary, uterus, gall bladder (Supplementary Figures S6A,F–I). To a lesser extent, the *Cdk13* was detected in the urinary bladder (Supplementary Figure S6D). Interestingly, rather localized, yet strong expression within organ structure was observed in renal pelvis in kidney, thyroid gland and heart atrium, with substantial expression in the heart ventricle (Supplementary Figures S6B,C,E).

### *Cdk13^*tm*1*d/tm*1*d*^* Mice Exhibits More Severe Phenotype and Earlier Lethality

**FIGURE 10:**
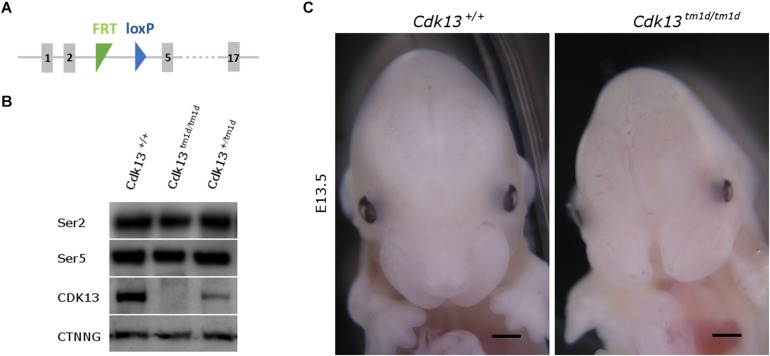
Generation of *Cdk13^*tm*1*d*^* mice. **(A)** Scheme of *Cdk13^*tm*1*d*^* allele harbors deletion of critical exons 3 and 4, causing non-functional allele of *Cdk13* gene. **(B)** Protein extract from the brain was prepared from E14.5 embryos and protein levels of CDK13 and γ-catenin (CTNNG) were evaluated by Western blotting. No residual expression of CDK13 was observed in *Cdk13^*tm*1*d/tm*1*d*^* embryos. Any significant downregulation of Ser2 and Ser5 phosphorylation was not observed in *Cdk13^*tm*1*d/tm*1*d*^* mice. **(C)** *Cdk13^*tm*1*d/tm*1*d*^* embryos exhibited distinct craniofacial phenotype with midfacial hypoplasia in comparison to wild-type embryos. Scale bar = 0.3 mm.

As we observed residual expression of *Cdk13* in analyzed organs of hypomorphic *Cdk13^*tm*1*a*/tm1*a*^* mice, we decided to cross *Cdk13^*tm*1*a*^* mice with Flp-deleter mice and Cre-deleter (detailed description of the utilized transgenic strains is provided at the Section “Experimental Procedure”) mice to generate *Cdk13^*tm*1*d*^* allele with deleted exons 3 and 4 (Figure 10A). As in case of *Cdk13^*tm*1*a*^* mice, the expression of CDK13 protein was investigated in the *Cdk13^*tm*1*d*^* mice. High expression of CDK13 was detected in the developing brain of WT *Cdk13*^+/+^ embryos (Figure 10B). Greatly downregulated expression of CDK13 was detected in heterozygous *Cdk13^*tm*1*d/+*^* brain with undetectable expression of CDK13 protein in homozygous *Cdk13^*tm*1/tm1*d*^* brain (Figure 10B). As in the case of *Cdk13^*tm*1*a*^* mice, no significant reproducible downregulation of Ser2 phosphorylation was observed in *Cdk13^*tm*1*d/tm*1*d*^* brain extract (Figure 10B). Interestingly, expected Mendelian ratios were reflected in the portion of *Cdk13^*tm*1*d/tm*1*d*^* mice. High number of empty decidua at E12.5 were detected reflecting increased mortality before this stage (Table 3). Critically, only 19 litters out of 42 contained *Cdk13^*tm*1*d/tm*1*d*^* embryos suggesting homozygous mice carrying *Cdk13^/tm1*d*^* alleles exhibited more severe defects in phenotype than *Cdk13^*tm*1*a*/tm1*a*^* mice, especially in craniofacial area with midline facial cleft (Figure 10C). The prevalence of the midline orofacial deficiency and pericardial effusion was 60.5% in *Cdk13^*tm*1*d*^* homozygous mice at E12.5-E14.5. Out of 94 cases, only two cases of *Cdk13^*tm*1*a/tm*1*a*^* mice had externally visible orofacial clefting (Supplementary Table S1). The prevalence of the pericardial effusion (PE) in *Cdk13^*tm*1*a/tm*1*a*^* embryos at E12.5-E14.5 was less than 20% in comparison to high occurrence in *Cdk13^*tm*1*d/tm*1*d*^* embryos (Supplementary Table S1). No PE or orofacial cleft was recorded in any WT, though 6 embryos from 262 heterozygotes exhibited PE.

**TABLE 3:** Genotypes of offspring from *Cdk13*^*tm1d/+*^ intercross.

					**Empty**
**Stage**	***Cdk13 ^+/+^***	***Cdk13*^tm1d/+^**	***Cdk13 ^*tm1d/tm1d*^***	**Litter**	**decidua**
	**(25%)**	**(50%)**	**(25%)**		
E12.5	33 (27.5%)	69 (57.5%)	18 (15%)^*,#^	18	24
E13.5	29 (31.5%)	52 (56.5%)	11 (12%)^*,#^	14	5
E14.5	24 (31.2%)	44 (57.1%)	9 (11.7%)^*,#^	10	4

^*^Growth retardation. ^#^Dead embryos.

### Comparison of *Cdk13^*tm*1*a/tm*1*a*^* and *Cdk13^*tm*1*d/tm*1*d*^* Mice at E13.5

**FIGURE 11:**
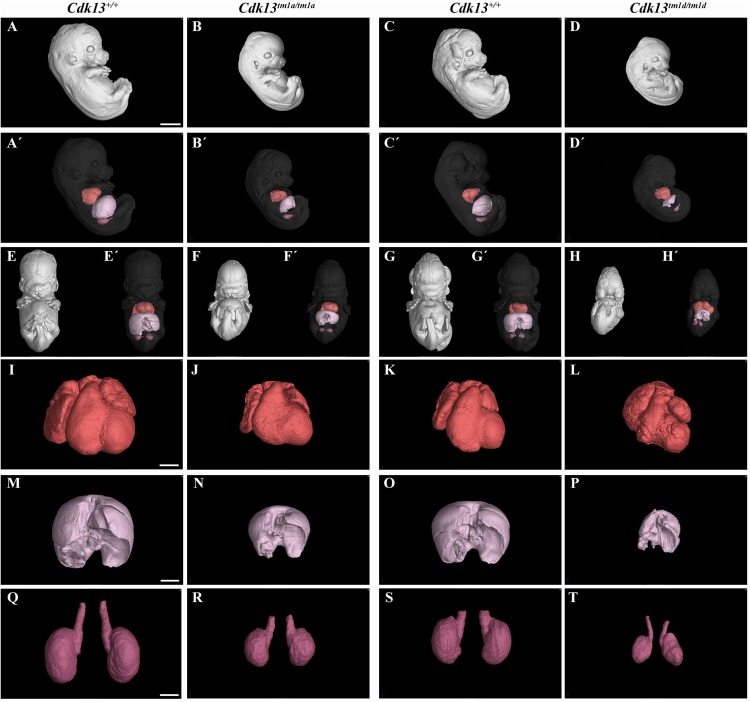
Comparison of *Cdk13^*tm*1*a*^* and *Cdk13^*tm*1*d*^* mice at E13.5 High-contrast differentiation resolution by X-ray computed microtomography where Lugol’s staining was used to visualize the soft tissues. The *Cdk13^*tm*1*a/tm*1*a*^*, *Cdk13^*tm*1*d/tm*1*d*^* and their littermate controls were analyzed at E13.5. **(A–D)** Overall view of *Cdk13*^+/+^, *Cdk13^*tm*1*a/tm*1*a*^*, and *Cdk13^*tm*1*d/tm*1*d*^* embryos. Both mutants exhibited smaller body size, with severe growth retardation in *Cdk13^*tm*1*d/tm*1*d*^* animals. **(A’–D’)** 3D reconstruction of heart (red color), kidney (violet color) and liver (light pink color) in right side view of embryo, with embryo outlined in gray where segmentation of serial sections was used for the reconstruction. Liver in *Cdk13^*tm*1*a/tm*1*a*^* and *Cdk13^*tm*1*d/tm*1*d*^* embryos were smaller than in *Cdk13*^+/+^ embryos. **(E–H’)** Frontal view of embryos with 3D reconstruction of heart, kidneys and liver. Clear deficiency in development of midfacial structures in *Cdk13^*tm*1*d/tm*1*d*^* embryos in contrast to *Cdk13*^+/+^ animals was observed. **(I–L)** 3D reconstruction of heart. The heart of *Cdk13^*tm*1*a/tm*1*a*^* and *Cdk13^*tm*1*d/tm*1*d*^* embryos were smaller in comparison to control mice with severe ventricle deficiency detected in *Cdk13^*tm*1*d/tm*1*d*^* animals. **(M–P)** 3D reconstruction of liver. Generally smaller liver were observed in *Cdk13^*tm*1*a/tm*1*a*^* and *Cdk13^*tm*1*d/tm*1*d*^* embryos in comparison to *Cdk13*^+/+^ littermate control mice. Liver in *Cdk13^*tm*1*d/tm*1*d*^* mice exhibit abrogated liver parts arrangements. **(Q–T)** 3D reconstruction of kidneys. Kidneys are smaller with abnormal shape in *Cdk13^*tm*1*a/tm*1*a*^* and *Cdk13^*tm*1*d/tm*1*d*^* embryos. Scale bar **(A–H)** = 1,5 mm, Scale bar **(I–L)** = 350 μm, scale bar **(M,P)** = 500 μm, and scale bar **(Q–T)** = 250 μm.

Finally, the phenotype of *Cdk13^*tm*1*a/tm*1*a*^* mice was compared to *Cdk13^*tm*1*d/tm*1*d*^* mice (Figure 11 and Supplementary Figures S7, S8). High-contrast differentiation resolution by X-ray computed microtomography was used to assess gross morphology of the *Cdk13*^+/+^, *Cdk13^*tm*1*a/tm*1*a*^* and *Cdk13^*tm*1*d/tm*1*d*^* embryos at E13.5. Both *Cdk13* mutants exhibited smaller body size with severe growth retardation in *Cdk13^*tm*1*d/tm*1*d*^* animals (Figures 11A–D). Hypoplasia of midfacial structures was observed in *Cdk13^*tm*1*d/tm*1*d*^* embryos (Figure 11, compare H to G). The hearts were smaller in both *Cdk13^*tm*1*a/tm*1*a*^* as well as *Cdk13^*tm*1*d/tm*1*d*^* embryos in comparison to littermate control mice with severe ventricle deficiency detected in *Cdk13^*tm*1*d/tm*1*d*^* animals (Figure 11, compare J to I and L to K). Generally smaller liver were detected in *Cdk13^*tm*1*a/tm*1*a*^* and *Cdk13^*tm*1*d/tm*1*d*^* embryos in comparison to control *Cdk13*^+/+^ mice. Liver in *Cdk13^*tm*1*d/tm*1*d*^* mice exhibited abrogated liver lobes arrangement (Figure 11, compare P to O and N to M). Kidneys in *Cdk13^*tm*1*d/tm*1*d*^* mice do not follow right left kidney asymmetry (Figure 11, compare T to S).

## Discussion

Our analysis revealed a new function of *Cdk13* in the context of the mouse development and found that this protein is critical for proper development at later stages of embryogenesis. Loss of *Cdk13* affects negatively several organs and tissue structures during mouse development and its inactivation leads to late embryonic lethality.

Animals with hypomorphic allele (*Cdk13^*tm*1*a*^*) retained low residual expression of CDK13 and they were lethal by E16.5 most due to heart failure and delayed development of several organs was caused likely due to insufficient supply of oxygen and nutrients. Interestingly, several miRNAs able to target *Cdk13* mRNA for degradation were recently recognized during the acute myocardial infarctions (Wang et al., 2015) supporting the idea of CDK13 as a strategic molecule for optimal heart function. In addition, the expression of CDK13 was detectable not just in the heart, but also in other organs during embryonic development, such as craniofacial area or brain. However, the expression only cannot explain the complexity of phenotype exclusively in tissue autonomous manner or by insufficient nutrients and oxygen distribution. Thus, we assume that the organs with higher expression might be affected either by non-functional CDK13 in cell autonomous manner and/or by underdevelopment of cardiovascular system and lack of nutrients, mostly by insufficient heart function. Importantly, the affected cardiovascular system is superior to development of other organ systems and can drive systemic developmental phenotype; the example of such phenotype is growth retardation in the whole embryo with no respect to local CDK13 expression levels, which is affecting size of all organs within the embryo body. Therefore, in organs where the expression of CDK13 is under detection limit, we suggest that the phenotype is caused mostly by failure of cardiovascular system development and its systemic influence.

It is also interesting why the *Cdk13^*tm*1*a*^* allele exhibits the hypomorphic mutant phenotype. One possible explanation might be due to the insertion of neomycin selection cassette in non-coding region that has been shown to affect gene expression, both at the DNA and RNA levels (Pham et al., 1996; Meyers et al., 1998; Scacheri et al., 2001). Moreover, *in silico* analyses of post-transcriptional exon shuffling (PTES) events in humans revealed high PTES frequencies in *CDK13* gene enabling the formation of aberrant functional CDK13 transcripts (Al-Balool et al., 2011).

In contrast to our observations, genetic depletion of *Cdk12*, a kinase with high amino acid similarity to *Cdk13*, resulted in early developmental lethality at the blastocyst stage due to deregulated expression of DNA-damage repair genes leading to enhanced genomic instability (Juan et al., 2016). Importantly, KO of CycK resembled the same lethal phenotype at the blastocyst stage as *Cdk12* animals (Blazek et al., 2011). Besides clear effect of CycK during preimplantation stage, CycK is highly expressed in mouse embryonic stem cells and testes in a developmentally regulated manner (Dai et al., 2012). During neonatal spermatogenesis, CycK is highly expressed in gonocytes, spermatogonial stem cells and is absent in differentiating spermatogonia, spermatids and spermatozoa (Xiang et al., 2014). Similar expression in testes, ovary and uterus was also documented for CDK13 in our heterozygous mice.

Downregulation of Cdk13 had also significant impact on brain development. It is known from previous studies that CycK, a Cdk13 binding partner, has been identified in a genome-wide screen as one of the factors involved in the formation of the nervous system in *Drosophila* (Neumuller et al., 2011). In parallel, function of CDK12 in process of embryonic neural development was described in the conditional KO mouse model for this gene (Chen et al., 2014, 2017). When neuronal differentiation model of mouse embryonic carcinoma cell (P19) was employed, CDK12 and CDK13 kinases participated in the axonal elongation through a common signaling pathway that modulates protein expression of CDK5 (Chen et al., 2014). Recently, CDK13 was found remarkably dysregulated in hippocampus and suggested as one of the hub genes useful to elucidate Alzheimer’s disease (Pang et al., 2017). Importantly, studies of patients with *CDK13* mutations suggested their essential role in brain development. As demonstrated in Figure 5, we were able to observe alteration of proliferation in neural tissue in *Cdk13^*tm*1*a*^* mice (as analyzed by Ki67 expression) however overall changes in brain morphology were not determined. On the other hand, observed proliferation changes can be also associated with developmental delay of *Cdk13^*tm*1*a*^* animals and subventricular zone in brains may significantly expands later in development. However, it is also possible that a prominent role of CDK13 may be apparent in later stages of brain development, for instance, in the axon pathfinding, synapse formation and etc. Confirmation of this statement, however, has to be proven in future by employing the nestin-Cre or CreErt-Flox systems.

Importantly, developmental defects within craniofacial formation was documented for *Cdk13^*tm*1*a*^* mice followed by complete loss of frontonasal part in *Cdk13^*tm*1*d*^* mice. It is probably the most surprising observation in respect to patients with heterozygous mutation of *CDK13* gene (Sifrim et al., 2016; Bostwick et al., 2017; Carneiro et al., 2018; Hamilton et al., 2018). Moreover, gradual deregulation of CDK13 in mice resulted in enhanced craniofacial phenotype. The hypomorphic *Cdk13^*tm*1*a*^* mice exhibited in the rostral area, altered shape of nasal septa and later at E15.5, incomplete formation of the secondary palate was observed in several animals, where palatal shelves did not reach the midline, while control animals exhibited complete fusion of palatal processes. Nevertheless, complete depletion of CDK13 in the *Cdk13^*tm*1*d/tm*1*d*^* mice led to ablation of the midline area of the head. There is a limited information regarding the role of CDK13 in this developmental process at the moment, yet. The association between SNP polymorphism rs373711932 located within *CDK13* gene and cranial base width was recently documented (Lee et al., 2017). Importantly, heterozygous copy number loss of *CCNK* gene in humans caused a syndromic neurodevelopmental disorder with distinctive facial dysmorphism (Fan et al., 2018). Therefore, it seems highly probable that CDK13 is indeed associated with craniofacial development, however, with unclear function, which will be necessary to uncover in the future.

From the functional point of view, it is surprising that only heterozygous mutation in one allele of the *CDK13* gene was sufficient to cause the CHDFIDD in human patients. Common feature in all studies depicting deleterious effect of pathogenic CDK13 in patients was presence of missense substitutions in the protein kinase domain around ATP-binding and magnesium binding sites (Sifrim et al., 2016; Bostwick et al., 2017; Hamilton et al., 2018). In addition, two nonsense variants and a frame-shift variant accompanying production of aberrant CDK13 transcripts have been identified (van den Akker et al., 2018). Based on crystal structure of CDK13/CycK complex, it is very possible that all these mutations most likely caused inactivation of catalytic activity of CDK13 or disrupted binding of CycK, its associating partner, leading to abrogated function of CDK13 (Kohoutek and Blazek, 2012; Greifenberg et al., 2016; Hamilton et al., 2018). Nevertheless, some of identified mutations in patients were predicted to retain binding to cyclin K with affected catalytic activity. If this is the case, then CDK13/CycK complex represents a dominant negative complex in cells (Hamilton et al., 2018). It is of note that two patients carrying stop codons at the end of the kinase domain represented milder phenotypes, although obvious genotype-phenotype correlation has not been recorded (van den Akker et al., 2018). From all these results, we can postulate that even loss of function of one *CDK13* allele is enough to interfere with developmental pathways and cause the CHDFIDD in humans, while complete abrogation of *CDK13* gene would lead to embryonic lethality as documented in our mouse model.

Currently, we can only speculate how CDK13 contributes to the developmental processes: if it participates in phosphorylation of Ser2 within CTD of RNAPII, it is highly probable that inhibition of CDK13 will block or dramatically affect ability of RNAPII to effectively synthesize nascent RNA. CDK13 was demonstrated to bind various splicing factors or proteins involved in splicing (Davidson et al., 2014; Bartkowiak and Greenleaf, 2015; Liang et al., 2015). Moreover, CDK13 was found to associate with ASF/SF2 factor, a member of the spliceosome complex (Even et al., 2006). CDK13 was also identified in perinucleolar compartments enriched for proteins primarily implicated in pre-mRNA processing; therefore, lack of CDK13 could abrogate also RNA processing of given metazoan genes (Even et al., 2016). Thus, it is possible that a loss of kinase activity or associating potential of CDK13 will cripple proper splicing of transcribed RNA by RNAPII resulting in production of improperly spliced mRNA of proteins engaged in development processes.

It has been postulated that CDK13 phosphorylates Ser2 and Ser5 within CTD of RNAPII, if Ser7 is prephosphorylated at the C terminus of heptapeptide (Greifenberg et al., 2016). However, we were not able to detect any effect on Ser2 or Ser5, either in *Cdk13^*tm*1*a/tm*1*a*^* or *Cdk13^*tm*1*d/tm*1*d*^* animals with substantial decreased level of CDK13 in developing brain, where physiologically high level of CDK13 is present. Even though the global effect on Ser2 or Ser5 was not documented in *Cdk13^*tm*1*a/tm*1*a*^* developing brains, CDK13 could still orchestrate phosphorylation of RNAPII at the specific sets of genes as earlier described for the CDK9/cyclin T2 or CDK12/cyclin K complexes in mouse and human cells (Zhu et al., 2009; Blazek et al., 2011; Johnson et al., 2016). When CDK13 was downregulated in HCT116 cells, gene ontology analysis revealed enrichment of functions connected to various extracellular and growth signaling pathways (Liang et al., 2015; Greifenberg et al., 2016). Similar *modus operandi* was implicated for CDK9 activation by MEK-1 and MSK1 in response to extracellular cues (Fujita et al., 2008; Smerdova et al., 2014). In addition, transcriptional kinase CDK8 was described to affect canonical Notch signaling by targeting activated *Notch* intracellular domain for proteasomal degradation (Fryer et al., 2004). In addition, CDK8 was suggested to stabilize β-catenin interaction with the promoter of WNT targets to achieve regulatory control (Firestein et al., 2008). Therefore, it is possible to envision that CDK13 regulates a specific set of genes at a specific developmental stage of embryonic development or in specialized cell types. Therefore, it will be critical in the near future to identify unique genes under control of CDK13 at given tissue or organ by employment of exome RNA-sequencing analyses in WT and *Cdk13*-deficient mice, which will be our next goal.

We believe that the *Cdk13^*tm*1*a*^* hypomorphic mouse can be a very instrumental animal model to delineate cellular pathways or mechanisms participating in the onset or propagation of severe developmental cues in humans with mutated form of CDK13. Moreover, our *Cdk13^*tm*1*d*^* mouse model with enhanced craniofacial phenotype may be invaluable for further clarification of molecular processes, which are responsible for clinical symptoms of human patients.

## Experimental Procedures

### Mice, Genotyping, Breeding of *Cdk13^*tm*1*a*^* Mice

Mouse embryos (*Cdk13^*tm*1*a(EUCOM)Hmgu*^*) bearing splicing acceptor, β-galactosidase and neomycine resistance gene both with poly A signals (Figure 1A) were obtained from the Infrafrontier Research Infrastructure–Mouse disease Models^1^. The acquired embryos carrying *Cdk13^*tm*1*a*^* allele were transferred into pseudopregnant females of C57BL/6N mouse strain and the heterozygous *Cdk13^*tm*1*a*^* mice were obtained at the Transgenic and Archiving Module, CCP (IMG, Prague, Czechia). Heterozygous mice for *Cdk13^*tm*1*a*^* allele were bred and newborn mice were genotyped by PCR amplification (94°C, 30 s; 62°C/65°C, 20 s; 72°C, 30 s; 35 cycles) of *Cdk13* gene to distinguish WT (*Cdk13*^+/+^) and *Cdk13^*tm*1*a/tm*1*a*^* alleles. For PCR analysis, the following sets of primers were used for KO and WT alleles: (KOf, 5′-CCA GCT TTA AAG GCG CAT AAC-3′; KOr, 5′-TGA CCT TGG GCA AGA ACA TAA-3′; WTf, 5′-TAG CTG GCC AAT GAG CTT TC-3′; WTr, 5′-AGT CTA GGA AGC TGG GAA GAT- 3′). Primers KOf and KOr amplify a 257-bp fragment from Cdk13^*tm*1*a*^ allele while primers WTf and WTr amplify a 179-bp fragment from WT allele. Genomic DNA was isolated from mouse ears by NaOH extraction (Truett et al., 2000). Heterozygous mice carrying *Cdk13^*tm*1*a*^* allele were bred and after mating, females were examined for vaginal plugs, embryonic day 0.5 (E0.5) was determined at noon at the day of vaginal plaque record. Embryos at various stages were obtained from uteri of pregnant heterozygous female mice, genotyped and the Mendelian ratios were defined.

To generate mouse carrying the *Cdk13^*tm*1*d*^* allele, the *Cdk13^*tm*1*a*/tm1*a*^* mice were crossed with the Flp-deleter mouse strain *Gt(ROSA)26Sor^*tm*2(CAG–flpo,–EYFP)Ics^* (MGI: 5285396, Philippe Soriano) and Cre-deleter strain *Gt(ROSA)26Sor^*tm*1(ACTB–cre,–EGFP)Ics^* (MGI: 5285392, Philippe Soriano) in order to delete critical exons 3 and 4 within *Cdk13* gene. All animal procedures were performed in strict accordance to the Guide for the Care and Use of Laboratory Animals and approved by the Institutional Animal Care and Use Committee (Veterinary Research Institute, Brno, Czechia).

### Western Blotting

Particular organ from *Cdk13^*tm*1*a/tm*1*a*^*, *Cdk13^*tm*1*a/+*^* and *Cdk13*^+/+^ embryos at E14.5 were lysed in lysis buffer (100 mM Tris, pH 7.4; 1% SDS; 10% glycerol) and sonicated. The brain from *Cdk13^*tm*1*d/tm*1*d*^*, *Cdk13^*tm*1*d/+*^* and *Cdk13*^+/+^ were lysed in lysis buffer (100 mM Tris, pH 7.4; 1% SDS; 10% glycerol) and sonicated. Concentrations of total proteins were determined with bicinchoninic acid (BCA). Lysates were diluted to the same concentration, mixed with the 3xLaemmli sample buffer and boiled for 5 min. Western blotting was performed using anti-mouse CDK13 polyclonal antibody (SAB1302350, Sigma-Aldrich), γ-catenin (CTNNG) antibody (2309, Cell Signaling Technology), cyclin T1 (CYCT1) (sc-10750, Santa Cruz Biotechnology) and anti-serine 2 (Ser2) and serine 5 (Ser5) rat monoclonal antibodies (clones 3E10 and 3E8 respectively, ChromoTek GmbH, Germany).

### Histological Analysis

Wild-type and homozygous *Cdk13^*tm*1*a/tm*1*a*^* embryos at E14.5 to E16.5 were fixed in 4% paraformaldehyde and dehydrated. After that hearts, brains, kidneys and livers were dissected, embedded in paraffin, and sectioned at 5 μm. Hematoxylin-eosin (H&E) staining was performed. Images were taken under bright field using a Leica compound microscope (DMLB2) with a Leica camera (DFC480) attached (Leica Microsystems, Wetzlar, Germany).

### Immunohistochemistry

Proliferating cells were visualized on E14.5 brain sections by labeling of Ki67 (positive cells are brown). Sections were pretreated in Citrate buffer, pH6, 10 mM, 20 min/97°C in water bath and were labeled with Ki67 primary antibody (RBK027-05, Zytomed Systems). For primary antibody detection, specific secondary antibody and avidin-biotin complex were used (Vectastain kit, PK-6101, Vector laboratories). The signal was developed by DAB chromogen system (K3468, DAKO). Nuclei were counterstained by hematoxylin (blue). Mitotic index was counted as the ratio between Ki67-positive cells and total amount of cells in three biological samples for each group (three *Cdk13*^+/+^ embryos, three *Cdk13^*tm*1*a/tm*1*a*^* embryos). Statistical significance in cell number differences between control and deficient mice were evaluated by unpaired *t*-test.

E14.5 heart sections were pretreated in DAKO Target Retrieval (32367, DAKO) solution for 15 min/97°C in water bath and labeled using primary antibodies against actin (sc-1615-R, Santa Cruz Biotechnology) and myosin (sc-32732, Santa Cruz Biotechnology). To detect primary antibodies, secondary Alexa Fluor antibodies (A11004, A11008, Invitrogen) were used. Nuclei were counterstained by DRAQ5TM (62251, Thermo Scientific). Images were captured on fluorescence confocal microscope Leica SP8 (Leica).

### Micro-Computed Tomography (micro-CT)

The embryos at E13.5 or E15.5 were contrasted with Lugol’s solution to visualize gross morphology of individual soft tissues by microCT. For the purpose of motion stabilization during the micro CT scan, mouse embryo was embedded in 1% agarose gel in Falcon conical centrifuge tube. The micro-CT scan was performed using - laboratory system GE Phoenix v| tome| x L 240 (GE Sensing & InspectionTechnologies GmbH, Germany), equipped with a 180 kV/15W maximum power nanofocus X-ray tube and high contrast flat panel detector DXR250 2048 px × 2048 px with 200 μm × 200 μm pixel size. The measurement was carried out in the air-conditioned cabinet (21°C) at acceleration voltage of 60 kV and X-ray tube current of 200 μA. Thousand nine hundred projections were taken over 360° with exposure time 900 ms resulting in 5.5 μm voxel resolution. The tomographic reconstruction was realized by software GE phoenix datos| x 2.0 (GE Sensing & Inspection Technologies GmbH, Germany). Reconstructed slice data were processed using VG Studio MAX 3.1 software (Volume Graphics GmbH, Germany) and segmentation of liver, kidneys and heart was completed manually.

### Embryonic Ultrasound Imaging and Doppler Echocardiography

Pregnant females were anesthetized on gestational day 14.5 or 15.5 (before noon) with an isoflurane/oxygen mixture (anesthesia was initiated with 3–4%, and maintained with 1–2% isoflurane), and maintained on a temperature-controlled mouse platform (with sensors for monitoring of maternal electrocardiogram, respiration and core body temperature). Maternal temperature was maintained at 34–37°C, and maternal heart beat at ∼400 beats/minute by adjusting the level of anesthesia. An incision of about 2–3 cm in the lower abdomen was made, and a uterine horn was externalized through the incision to provide imaging access. The number of fetuses in right and left uterine horns was counted from the mother’s bladder and later during echocardiographic imaging, labeled as R1, R2, R3, etc (right side) and L1, L2, L3, etc (left side). Pre-warmed ultrasound gel (37°C) without bubbles was applied between the individual fetuses and the transducer. B-mode imaging scanning of the whole embryo with the focus on the heart and Color Doppler and PW Doppler measurements for heart aorta and dorsal aorta were obtained using a high-frequency ultrasound system (Vevo 2100, FUJIFILM VisualSonics, Inc., Toronto, ON, Canada) equipped with a MS-550S transducer operating at a center frequency of 44 MHz. At the end of imaging of all fetuses, the mother was sacrificed by cervical dislocation and a small piece of yolk sac of each fetus was taken for genotyping.

## Data Availability

All datasets generated for this study are included in the manuscript and/or the Supplementary Files.

## Ethics Statement

All animal procedures were performed in strict accordance to the Guide for the Care and Use of Laboratory Animals and approved by the Institutional Animal Care and Use Committee (Veterinary Research Institute, Brno, Czechia).

## Author Contributions

JKo conceptualized and supervised the project, acquired the funding, wrote the original draft, and administrated the project. MN conceptualized the project, contributed to formal analysis, investigation, and visualization, and wrote, review, and edited the manuscript. DV contributed to investigation and visualization. MH contributed to formal analysis, investigation, and visualization. JP contributed to conceptualization, formal analysis, supervision, and investigation and wrote, review, and edited the manuscript. SP and MP contributed to investigation and visualization. RS supervised the project. MK, TZ, and JKa contributed to software development and visualization. H-CJ contributed to investigation, and wrote, review, and edited the manuscript. M-JF and MB contributed to formal analysis, supervision, writing, review, and editing.

## Conflict of Interest Statement

The authors declare that the research was conducted in the absence of any commercial or financial relationships that could be construed as a potential conflict of interest.

## Footnotes

1 https://www.infrafrontier.eu/

## Supplementary Material

FIGURE S1 Truncated form of CDK13 protein. **(A)**Predicted amino acid sequence of truncated CDK13 protein due totranscriptional block mediated by two Poly A sites introduced intothe intron 2 as part of trap vector in mice bearing *Cdk13^*tm*1*a*^* allele.Black and Blue capital letters represent amino acids encoded within Exon 1 and Exon 2 of *Cdk13* gene, respectively. Predicted Molecular weight of truncated *CDK13* protein is 66.22 KDa (https://www.bioinformatics.org/sms/ prot_mw.html). **(B)** Brain extracts were prepared from E14.5 embryos of given genotype and protein levels of wild-type and truncated form of CDK13 was evaluated by Western Blotting. The antibody used throughout our study recognizes N-terminal part of CDK13, which is expressed in both forms of Cdk13, wild-type as well as truncated one. The *Cdk13*^+/+^ mice contained only the wild-type CDK13 protein (WT-CDK13). The heterozygous *Cdk13^*tm*1*a/+*^* mice included wild-type (WT-CDK13) and truncated (TR-CDK13) forms of CDK13 protein. The *Cdk13^*tm*1*a*^* expressed the truncated form of CDK13 and minor wild-type CDK13 as demonstrated by immunoblot.

FIGURE S2 Comparison of gross morphology of *Cdk13*-deficent mice phenotype. **(A–H)** Wild-type *Cdk13*^+/+^, **(I–P)** heterozygous *Cdk13^*tm*1*a/+*^*, and **(Q–X)** *Cdk13^*tm*1*a*^* embryos at various stages. Growth retardation of *Cdk13*-deficient mice was apparent from early developmental stages **(Q,R)**. Some embryos exhibited nuchal edema and peripheral hypervascularization **(U)** and abnormal craniofacial shape **(S,T)**. Developmental delay was observed at later stages **(V,W)**. At E16.5, each of the *Cdk13^*tm*1*a/tm*1*a*^* embryos was undergoing resorption **(X)**. No morphological differences between wild-type and heterozygous embryos were observed. Scale bar = 0.1 mm.

FIGURE S3 The 3D reconstruction of selected organs in micro-CT of control *Cdk13*^+/+^ and *Cdk13^*tm*1*a/tm*1*a*^* embryos. Overall view on embryos of *Cdk13*^+/+^ **(A)** and *Cdk13^*tm*1*a/tm*1*a*^* **(B)** with 3D reconstruction of heart, liver and kidney (left). Gross morphology is well visible on movable model on the right side. To visualize only one organ, click on the lower row and select heart (orange), liver (pink) or kidney (purple) organ only. Scale bar = 1 mm.

FIGURE S4 Transversal sections of the head of control *Cdk13*^+/+^ and *Cdk13^*tm*1*a/tm*1*a*^* embryos. Transversal sections through the mouse head with details of the nasal cavity area. At E14.5, no obvious differences were observed and only one epithelial protrusion (marked by arrow) were developed close to each nasal cavity (nc) (compare **A** to **D**). Later at E15.5, the number of nasal glands was reproducibly smaller in *Cdk13^*tm*1*a/tm*1*a*^* mice in comparison to control animals (compare **B** to **E**). **(C,F)** Coronal section through the head of embryos at the level of the eyes. White arrows depict different complexity of the nasal cavity in wild-type, *Cdk13*^+/+^, and *Cdk13^*tm*1*a/tm*1*a*^* animals. Abbreviations nc – nasal cavity, arrowhead – nasal gland. Scale bar **(A,B,D,F)** = 100 μm and scale bar **(C,F)** = 1 mm.

FIGURE S5 Immunohistochemical detection of myosin and actin in the heart of *Cdk13*^+/+^ and *Cdk13^*tm*1*a/tm*1*a*^* embryos. Sagittal sections through the mouse heart were prepared and immunohistochemical detection of myosin and actin was carried out with specific antibodies. Decreased expression of myosin was detected in ventricle myocardium, compare B and B’ to E and E’. Scale bar **(A,D)** = 500 μm; Scale bar (**B–F**, without D) − 100 μm.

FIGURE S6 The expression of *Cdk13* in adult mouse organs visualized by β-galactosidase activity. The expression of *Cdk13* was examined by activity of β-galactosidase in adult mouse organs **(A)** eye, **(B)** heart, **(C)** thyroid gland, **(D)** urinary bladder, **(E)** kidney, **(F)** uterus, **(G)** gall bladder, **(H)** testes, and **(I)** ovary.

FIGURE S7 Sagittal sections through heads of *Cdk13*^*t**m**a*1^ and *Cdk13*^*t**m*1*d*^ and *Cdk13*^*t**m**a*1^ and *Cdk13*^*t**m*1*d*^ mice. High-contrast differentiation resolution by X-ray computed microtomography where Lugol’s staining was used to visualize the soft tissues. The *Cdk13^*tm*1*a/tm*1*a*^*, *Cdk13^*tm*1*d/tm*1*d*^* and their littermate controls are displayed at E13.5 to show overall developmental delay.

FIGURE S8 Horizontal sections through heads of *Cdk13*^*t**m**a*1^ and *Cdk13*^*t**m*1*d*^ mice. High-contrast differentiation resolution by X-ray computed microtomography where Lugol’s staining was used to visualize the soft tissues. The *Cdk13^*tm*1*a/tm*1*a*^*, *Cdk13^*tm*1*d/tm*1*d*^* and their littermate controls are displayed at E13.5.

TABLE S1 The prevalence of developmental defects in *Cdk13*^*t**m**a*1^ and *Cdk13*^*t**m*1*d*^ transgenic mice.
