# Supplementary material for: Mouse Model of Congenital Heart Defects, Dysmorphic Facial Features and Intellectual Developmental Disorders as a Result of Non-functional CDK13
Source: Front Cell Dev Biol. 2019 Aug 7;7:155. doi: 10.3389/fcell.2019.00155 (PMC6694211; doi:10.3389/fcell.2019.00155)
Supplement: FIGURE S1 — Truncated form of CDK13 protein. (A)Predicted amino acid sequence of truncated CDK13 protein due totranscriptional block mediated by two Poly A sites introduced intothe intron 2 as part of trap vector in mice bearing Cdk13tm1a allele.Black and Blue capital letters represent amino acids encoded within Exon 1 and Exon 2 of Cdk13 gene, respectively. Predicted Molecular weight of truncated CDK13 protein is 66.22 KDa (https://www.bioinformatics.org/sms/ prot_mw.html). (B) Brain extracts were prepared from E14.5 embryos of given genotype and protein levels of wild-type and truncated form of CDK13 was evaluated by Western Blotting. The antibody used throughout our study recognizes N-terminal part of CDK13, which is expressed in both forms of Cdk13, wild-type as well as truncated one. The Cdk13+/+ mice contained only the wild-type CDK13 protein (WT-CDK13). The heterozygous Cdk13tm1a/+ mice included wild-type (WT-CDK13) and truncated (TR-CDK13) forms of CDK13 protein. The Cdk13tm1a expressed the truncated form of CDK13 and minor wild-type CDK13 as demonstrated by immunoblot. [file Presentation_1.PPT]

## Slide 1
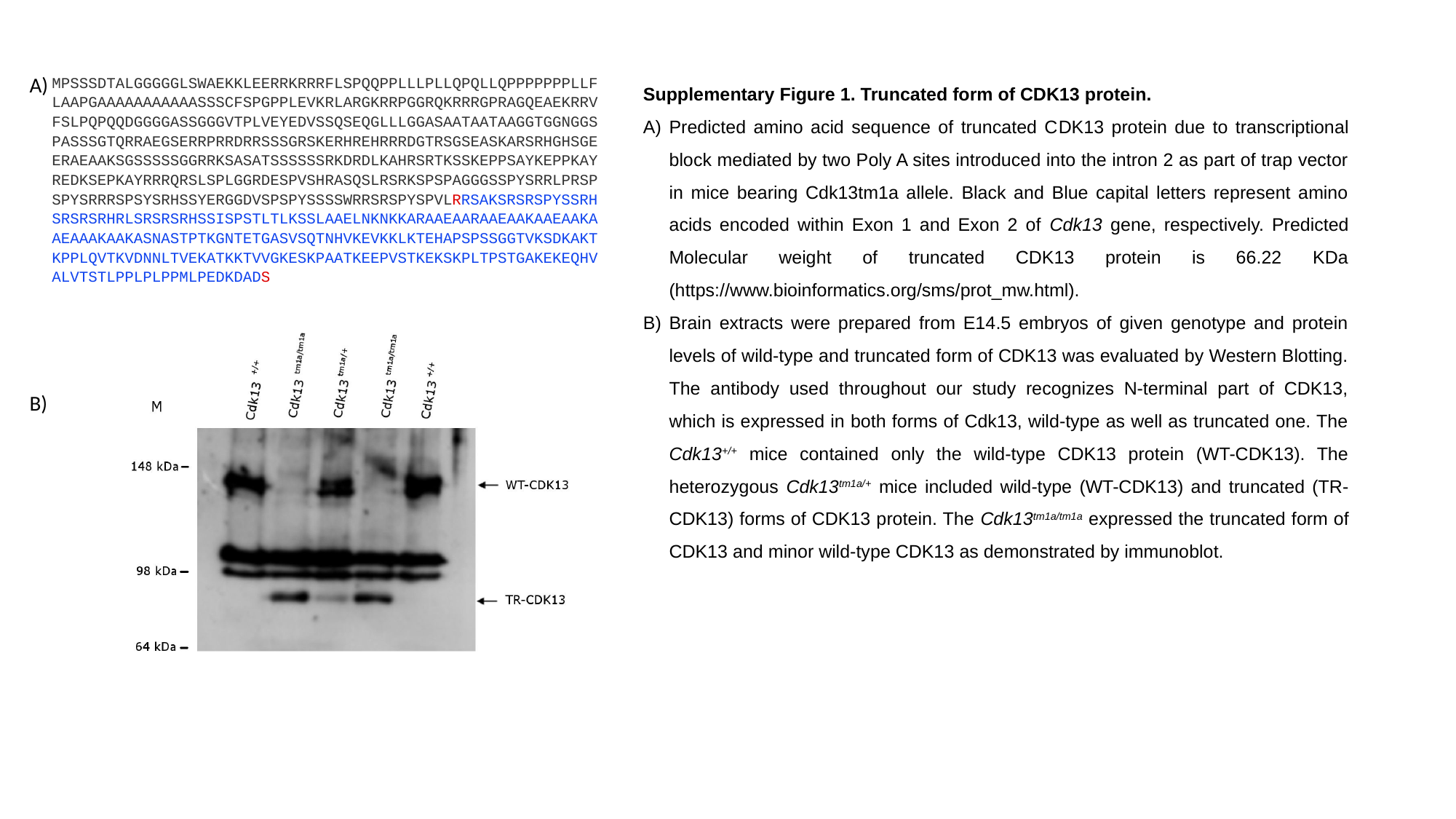

A)
MPSSSDTALGGGGGLSWAEKKLEERRKRRRFLSPQQPPLLLPLLQPQLLQPPPPPPPLLF
LAAPGAAAAAAAAAAASSSCFSPGPPLEVKRLARGKRRPGGRQKRRRGPRAGQEAEKRRV
FSLPQPQQDGGGGASSGGGVTPLVEYEDVSSQSEQGLLLGGASAATAATAAGGTGGNGGS
PASSSGTQRRAEGSERRPRRDRRSSSGRSKERHREHRRRDGTRSGSEASKARSRHGHSGE
ERAEAAKSGSSSSSGGRRKSASATSSSSSSRKDRDLKAHRSRTKSSKEPPSAYKEPPKAY
REDKSEPKAYRRRQRSLSPLGGRDESPVSHRASQSLRSRKSPSPAGGGSSPYSRRLPRSP
SPYSRRRSPSYSRHSSYERGGDVSPSPYSSSSWRRSRSPYSPVLRRSAKSRSRSPYSSRH
SRSRSRHRLSRSRSRHSSISPSTLTLKSSLAAELNKNKKARAAEAARAAEAAKAAEAAKA
AEAAAKAAKASNASTPTKGNTETGASVSQTNHVKEVKKLKTEHAPSPSSGGTVKSDKAKT
KPPLQVTKVDNNLTVEKATKKTVVGKESKPAATKEEPVSTKEKSKPLTPSTGAKEKEQHV
ALVTSTLPPLPLPPMLPEDKDADS
Supplementary Figure 1. Truncated form of CDK13 protein.
Predicted amino acid sequence of truncated CDK13 protein due to transcriptional block mediated by two Poly A sites introduced into the intron 2 as part of trap vector in mice bearing Cdk13tm1a allele. Black and Blue capital letters represent amino acids encoded within Exon 1 and Exon 2 of Cdk13 gene, respectively. Predicted Molecular weight of truncated CDK13 protein is 66.22 KDa (https://www.bioinformatics.org/sms/prot_mw.html).
Brain extracts were prepared from E14.5 embryos of given genotype and protein levels of wild-type and truncated form of CDK13 was evaluated by Western Blotting. The antibody used throughout our study recognizes N-terminal part of CDK13, which is expressed in both forms of Cdk13, wild-type as well as truncated one. The Cdk13+/+ mice contained only the wild-type CDK13 protein (WT-CDK13). The heterozygous Cdk13tm1a/+ mice included wild-type (WT-CDK13) and truncated (TR-CDK13) forms of CDK13 protein. The Cdk13tm1a/tm1a expressed the truncated form of CDK13 and minor wild-type CDK13 as demonstrated by immunoblot.
B)

## Slide 2
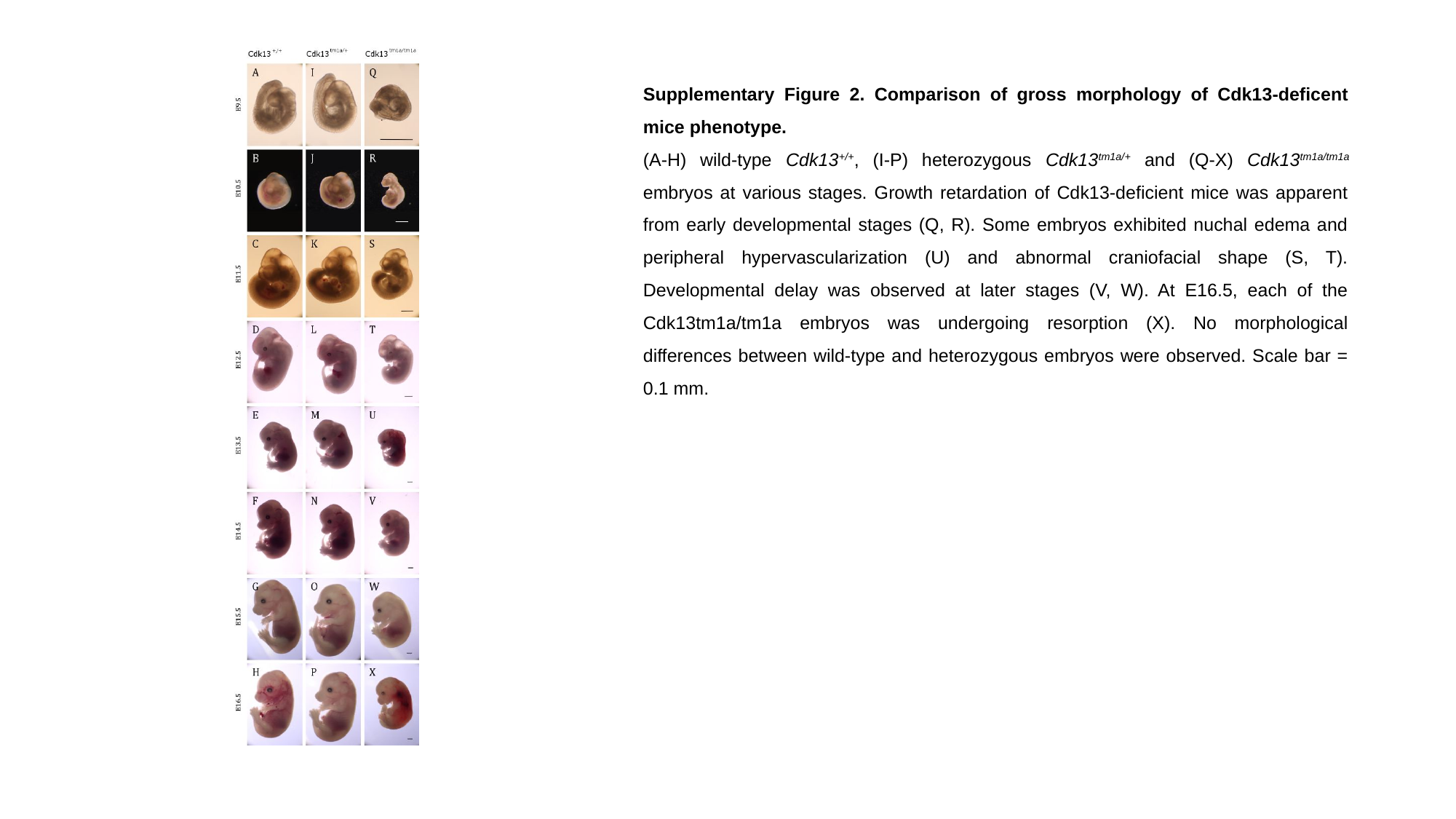

Supplementary Figure 2. Comparison of gross morphology of Cdk13-deficent mice phenotype.
(A-H) wild-type Cdk13+/+, (I-P) heterozygous Cdk13tm1a/+ and (Q-X) Cdk13tm1a/tm1a embryos at various stages. Growth retardation of Cdk13-deficient mice was apparent from early developmental stages (Q, R). Some embryos exhibited nuchal edema and peripheral hypervascularization (U) and abnormal craniofacial shape (S, T). Developmental delay was observed at later stages (V, W). At E16.5, each of the Cdk13tm1a/tm1a embryos was undergoing resorption (X). No morphological differences between wild-type and heterozygous embryos were observed. Scale bar = 0.1 mm.

## Slide 3
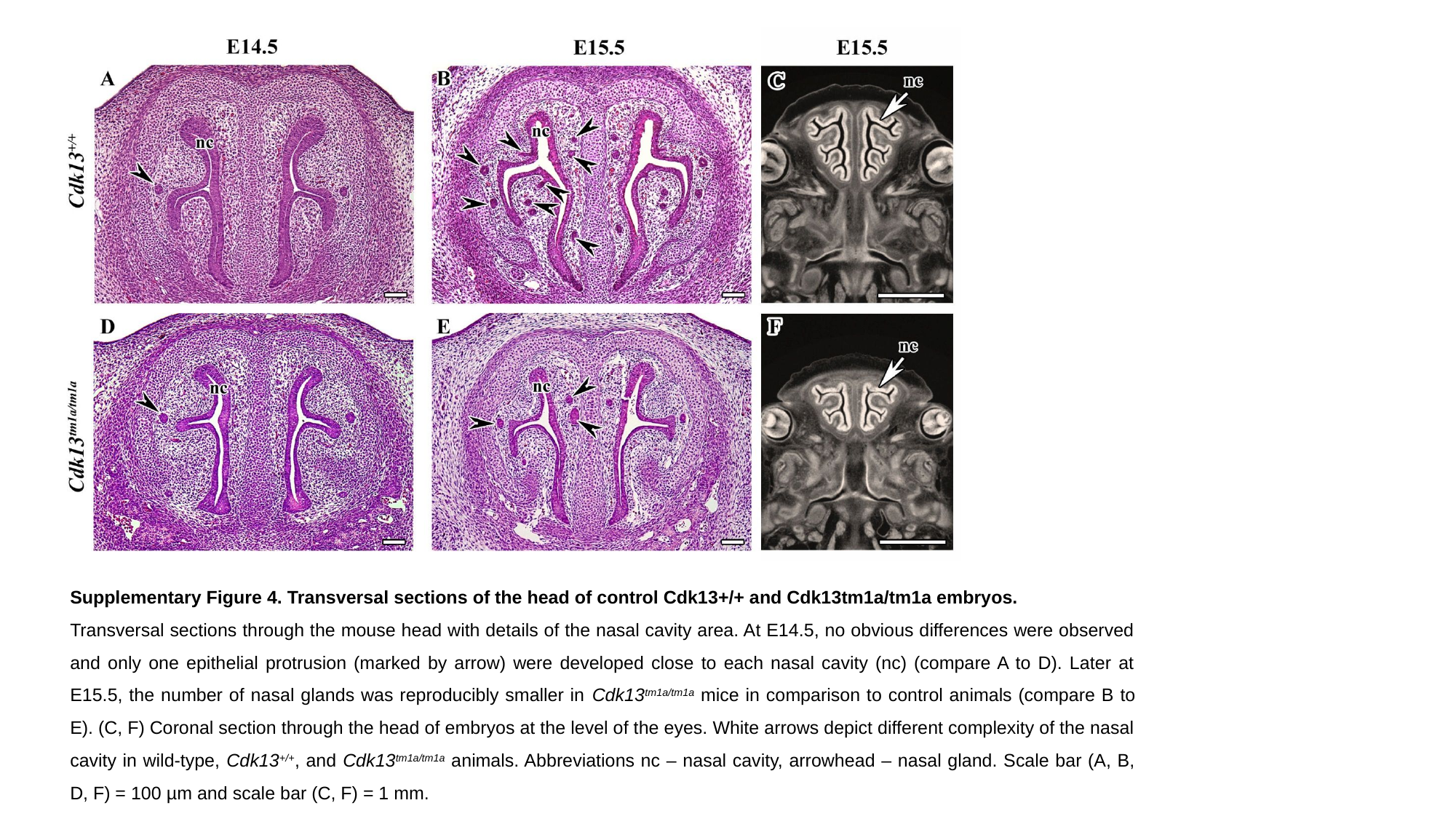

Supplementary Figure 4. Transversal sections of the head of control Cdk13+/+ and Cdk13tm1a/tm1a embryos.
Transversal sections through the mouse head with details of the nasal cavity area. At E14.5, no obvious differences were observed and only one epithelial protrusion (marked by arrow) were developed close to each nasal cavity (nc) (compare A to D). Later at E15.5, the number of nasal glands was reproducibly smaller in Cdk13tm1a/tm1a mice in comparison to control animals (compare B to E). (C, F) Coronal section through the head of embryos at the level of the eyes. White arrows depict different complexity of the nasal cavity in wild-type, Cdk13+/+, and Cdk13tm1a/tm1a animals. Abbreviations nc – nasal cavity, arrowhead – nasal gland. Scale bar (A, B, D, F) = 100 µm and scale bar (C, F) = 1 mm.

## Slide 4
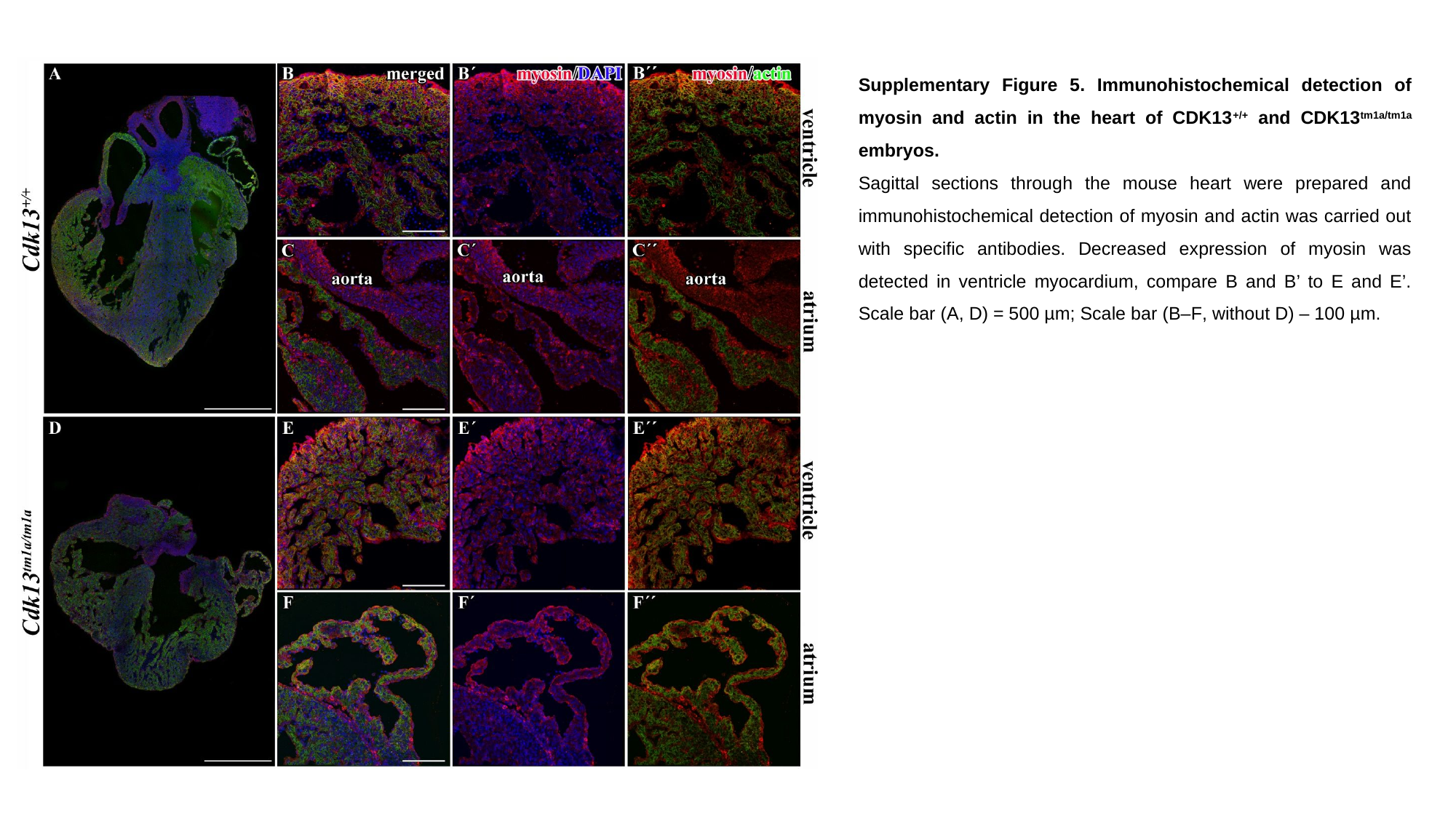

Supplementary Figure 5. Immunohistochemical detection of myosin and actin in the heart of CDK13+/+ and CDK13tm1a/tm1a embryos.
Sagittal sections through the mouse heart were prepared and immunohistochemical detection of myosin and actin was carried out with specific antibodies. Decreased expression of myosin was detected in ventricle myocardium, compare B and B’ to E and E’. Scale bar (A, D) = 500 µm; Scale bar (B–F, without D) – 100 µm.

## Slide 5
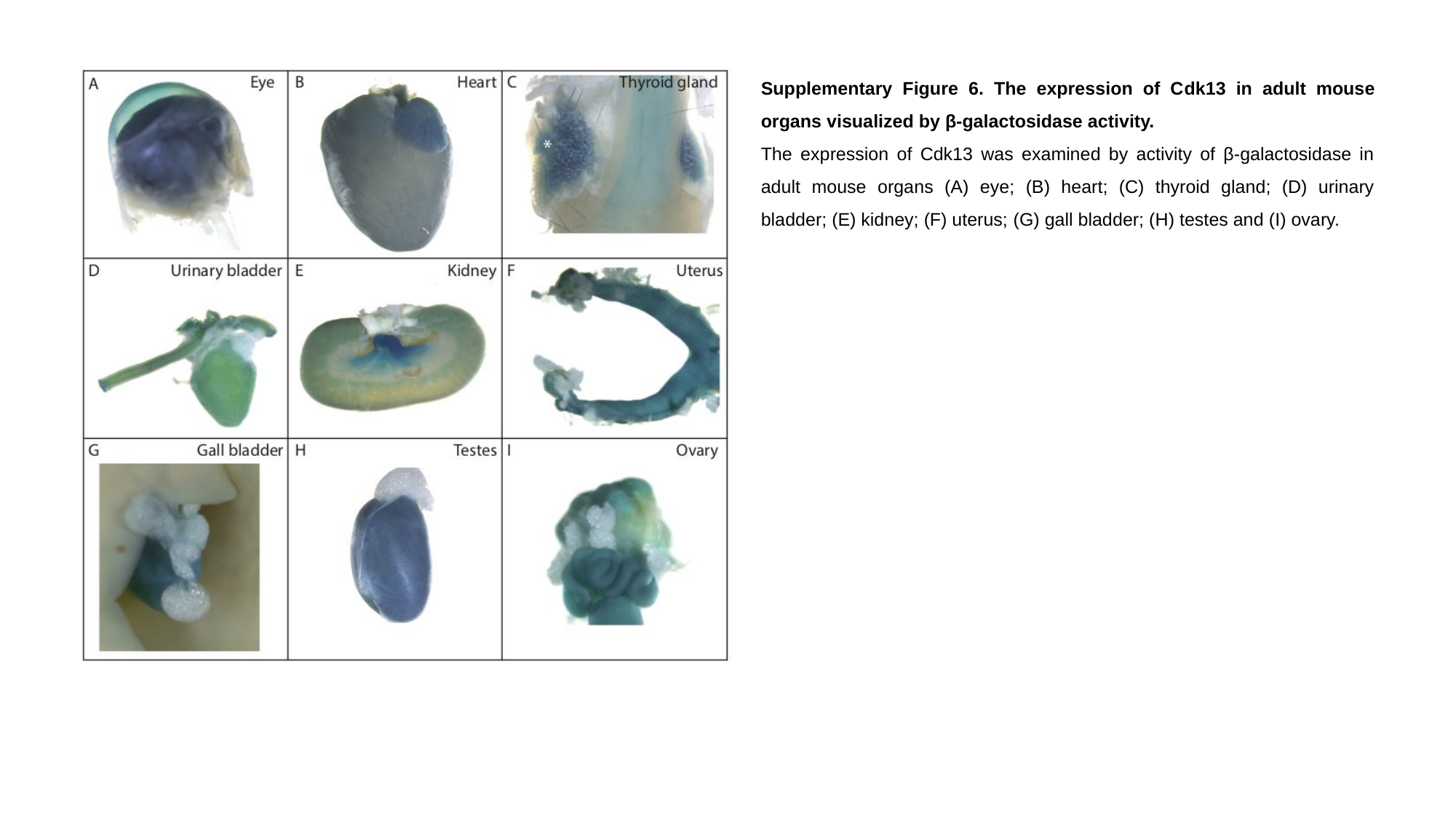

Supplementary Figure 6. The expression of Cdk13 in adult mouse organs visualized by β-galactosidase activity.
The expression of Cdk13 was examined by activity of β-galactosidase in adult mouse organs (A) eye; (B) heart; (C) thyroid gland; (D) urinary bladder; (E) kidney; (F) uterus; (G) gall bladder; (H) testes and (I) ovary.

## Slide 6
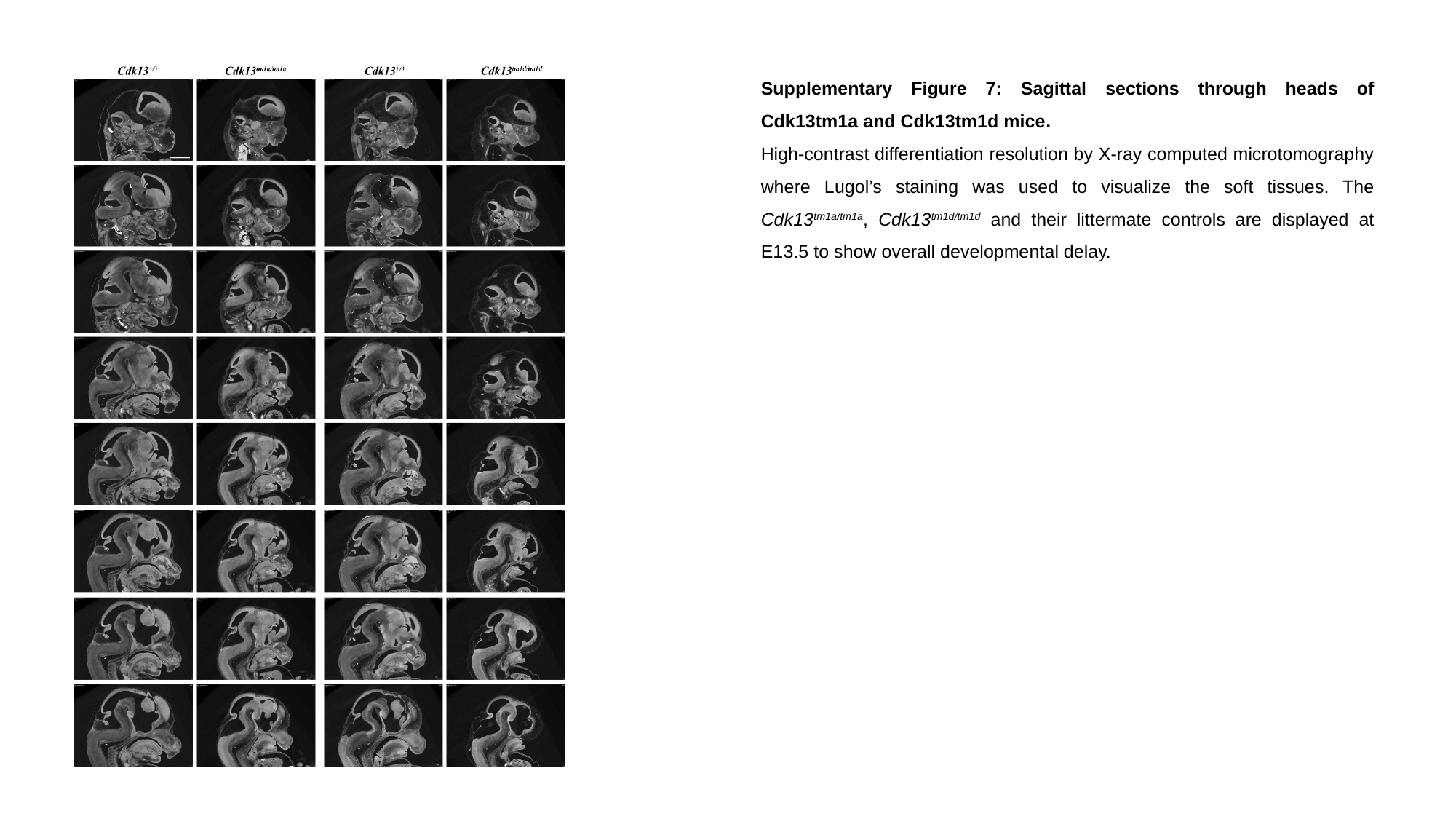

Supplementary Figure 7: Sagittal sections through heads of Cdk13tm1a and Cdk13tm1d mice.
High-contrast differentiation resolution by X-ray computed microtomography where Lugol’s staining was used to visualize the soft tissues. The Cdk13tm1a/tm1a, Cdk13tm1d/tm1d and their littermate controls are displayed at E13.5 to show overall developmental delay.

## Slide 7
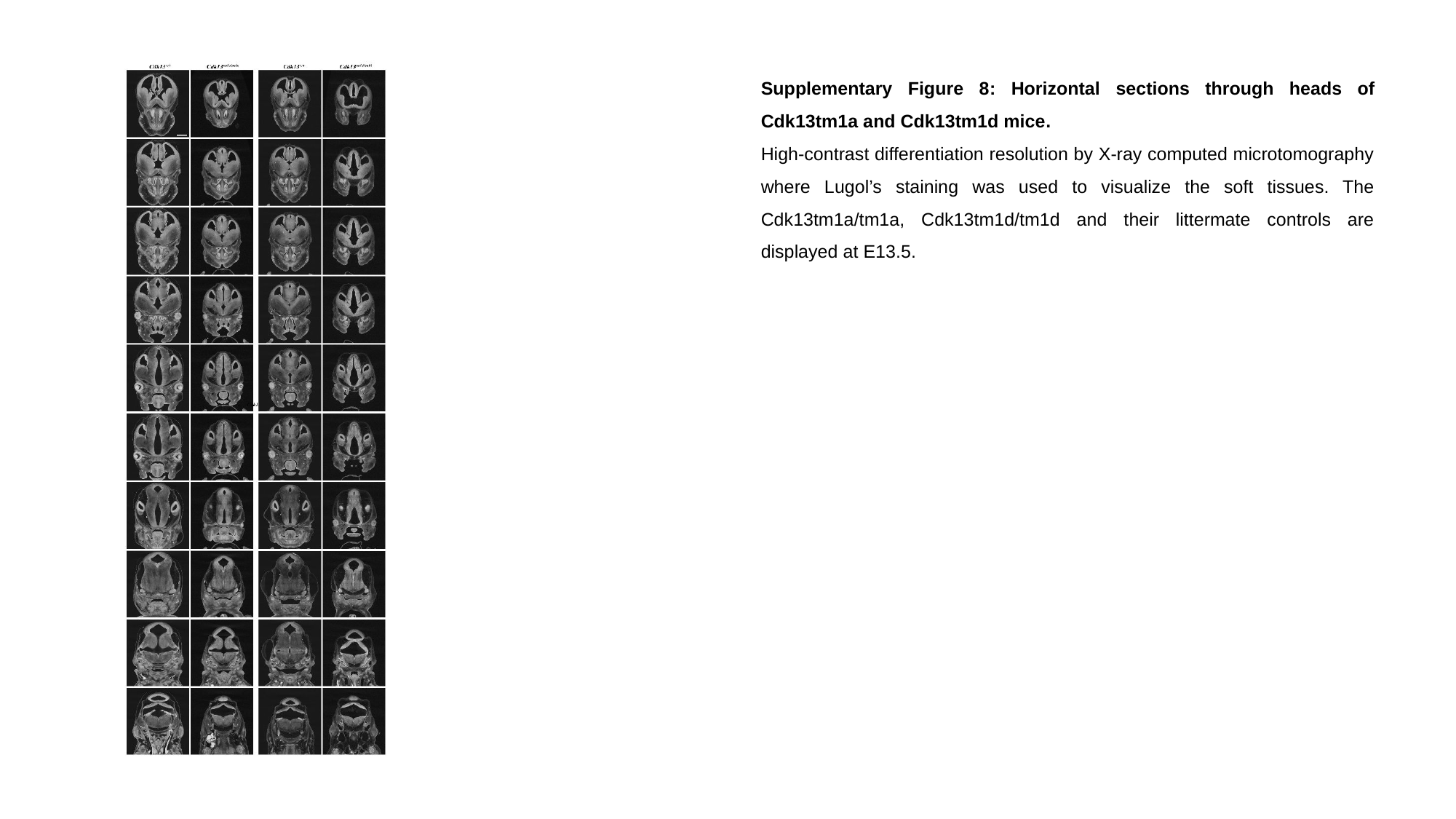

Supplementary Figure 8: Horizontal sections through heads of Cdk13tm1a and Cdk13tm1d mice.
High-contrast differentiation resolution by X-ray computed microtomography where Lugol’s staining was used to visualize the soft tissues. The Cdk13tm1a/tm1a, Cdk13tm1d/tm1d and their littermate controls are displayed at E13.5.

## Slide 8
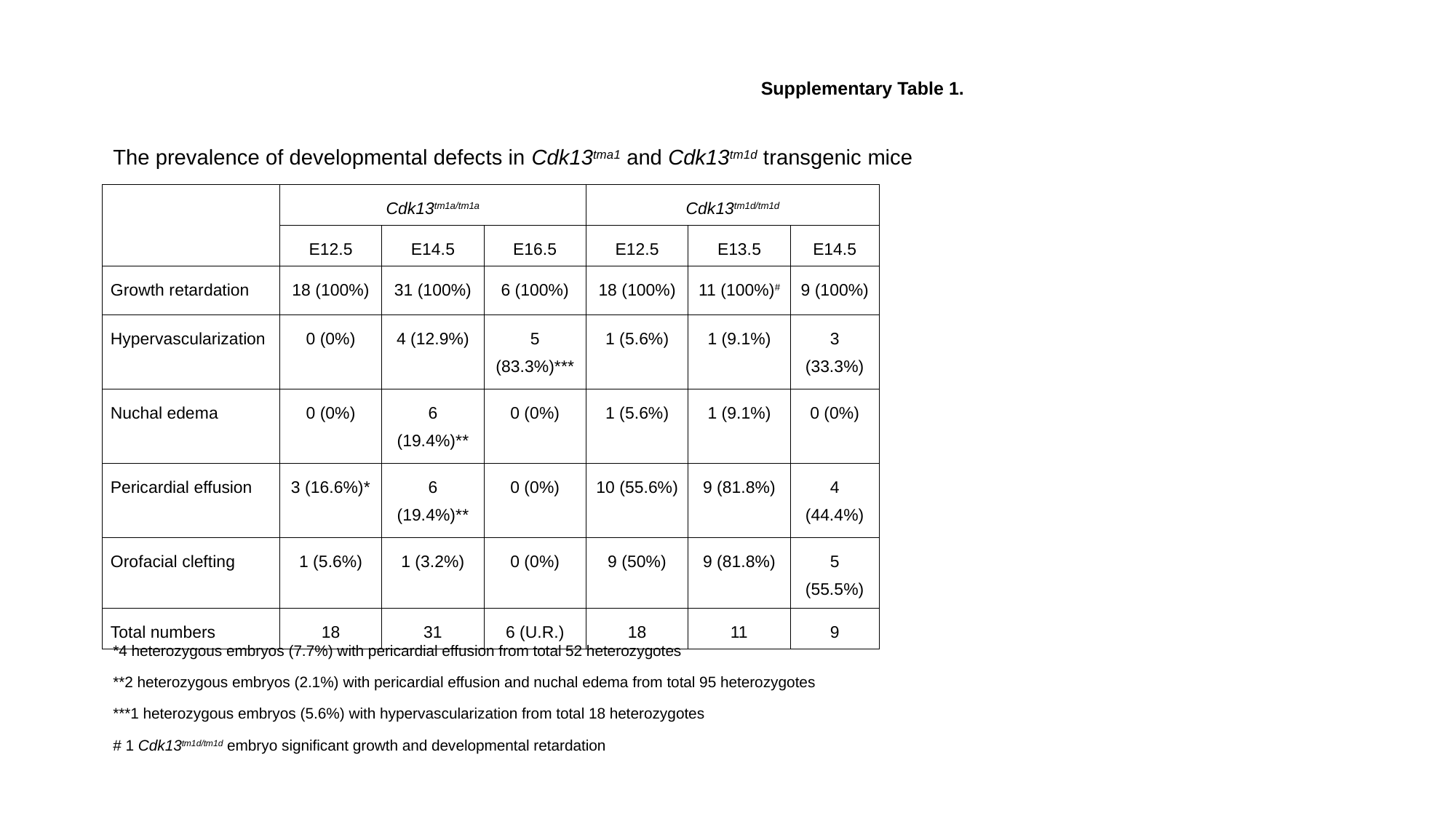

Supplementary Table 1.
The prevalence of developmental defects in Cdk13tma1 and Cdk13tm1d transgenic mice
| | Cdk13tm1a/tm1a | | | Cdk13tm1d/tm1d | | |
| --- | --- | --- | --- | --- | --- | --- |
| | E12.5 | E14.5 | E16.5 | E12.5 | E13.5 | E14.5 |
| Growth retardation | 18 (100%) | 31 (100%) | 6 (100%) | 18 (100%) | 11 (100%)# | 9 (100%) |
| Hypervascularization | 0 (0%) | 4 (12.9%) | 5 (83.3%)\*\*\* | 1 (5.6%) | 1 (9.1%) | 3 (33.3%) |
| Nuchal edema | 0 (0%) | 6 (19.4%)\*\* | 0 (0%) | 1 (5.6%) | 1 (9.1%) | 0 (0%) |
| Pericardial effusion | 3 (16.6%)\* | 6 (19.4%)\*\* | 0 (0%) | 10 (55.6%) | 9 (81.8%) | 4 (44.4%) |
| Orofacial clefting | 1 (5.6%) | 1 (3.2%) | 0 (0%) | 9 (50%) | 9 (81.8%) | 5 (55.5%) |
| Total numbers | 18 | 31 | 6 (U.R.) | 18 | 11 | 9 |
*4 heterozygous embryos (7.7%) with pericardial effusion from total 52 heterozygotes
**2 heterozygous embryos (2.1%) with pericardial effusion and nuchal edema from total 95 heterozygotes
***1 heterozygous embryos (5.6%) with hypervascularization from total 18 heterozygotes
# 1 Cdk13tm1d/tm1d embryo significant growth and developmental retardation
