# Supplementary figures and images for: Mouse Model of Congenital Heart Defects, Dysmorphic Facial Features and Intellectual Developmental Disorders as a Result of Non-functional CDK13
Source: Front Cell Dev Biol. 2019 Aug 7;7:155. doi: 10.3389/fcell.2019.00155 (PMC6694211; doi:10.3389/fcell.2019.00155)

A

# Cdk 13 wt

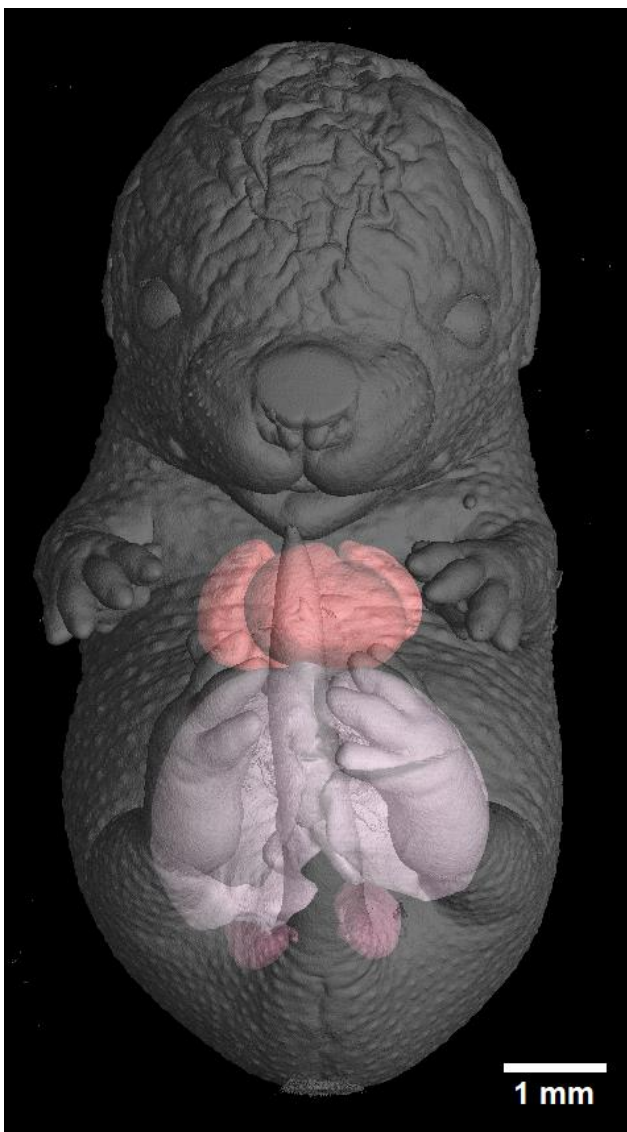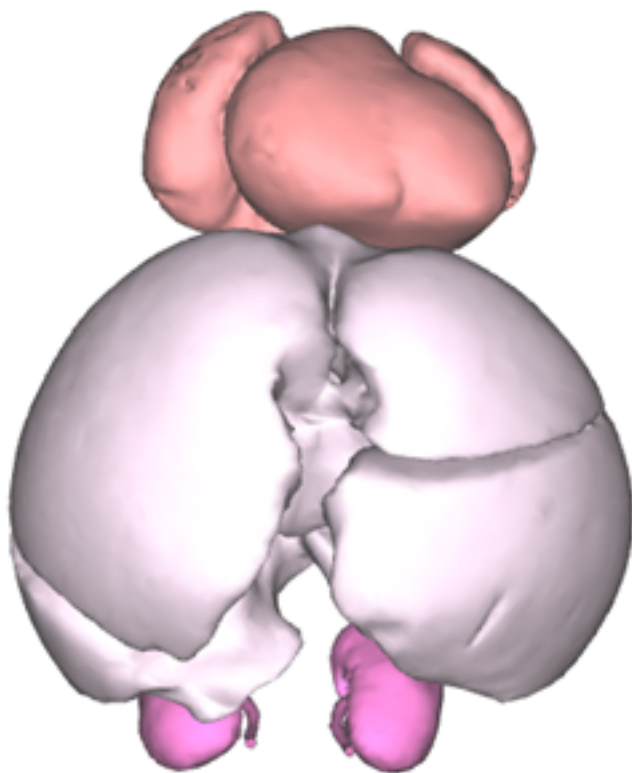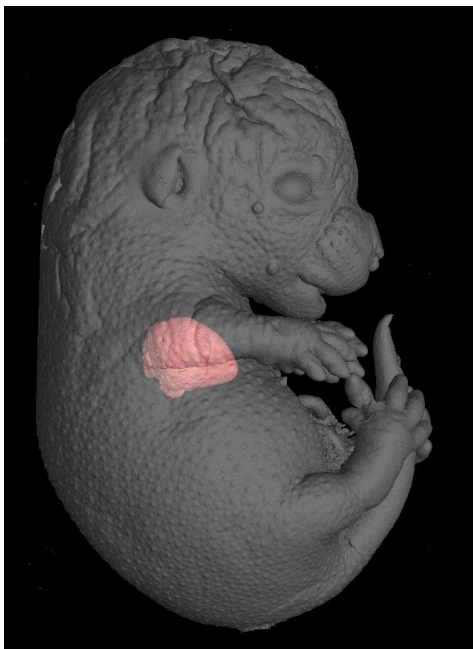

HEART

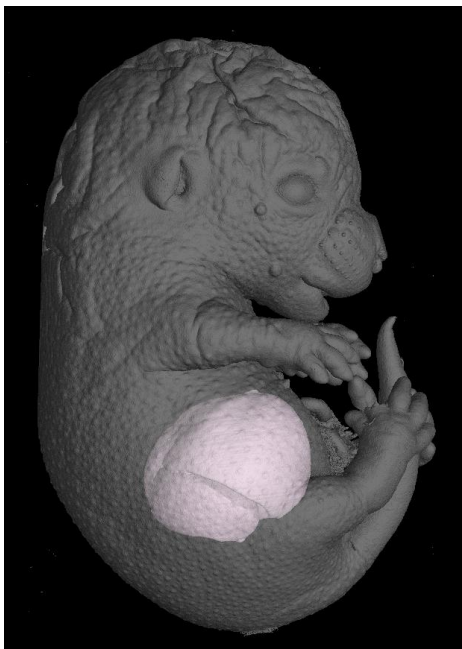

LIVER

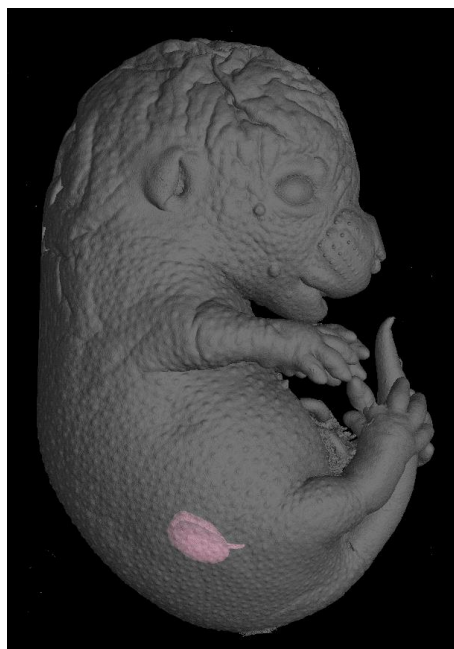

KIDNEYS

B

# Cdk 13 KO

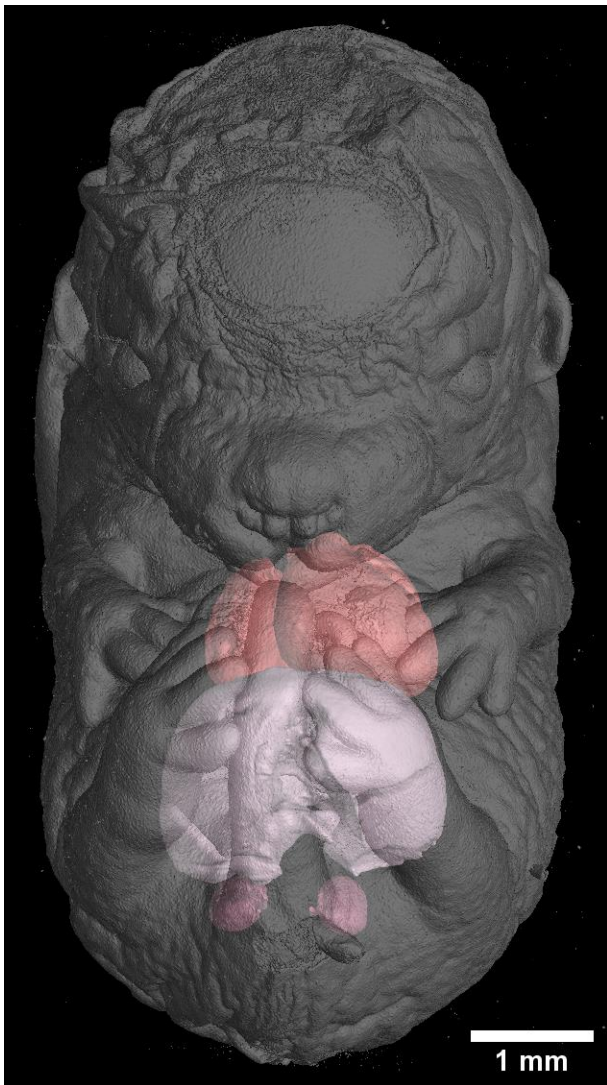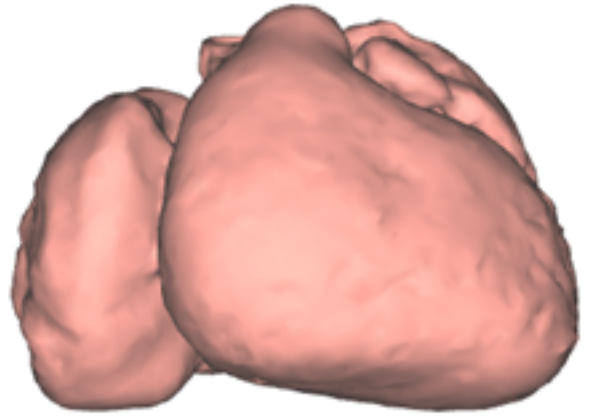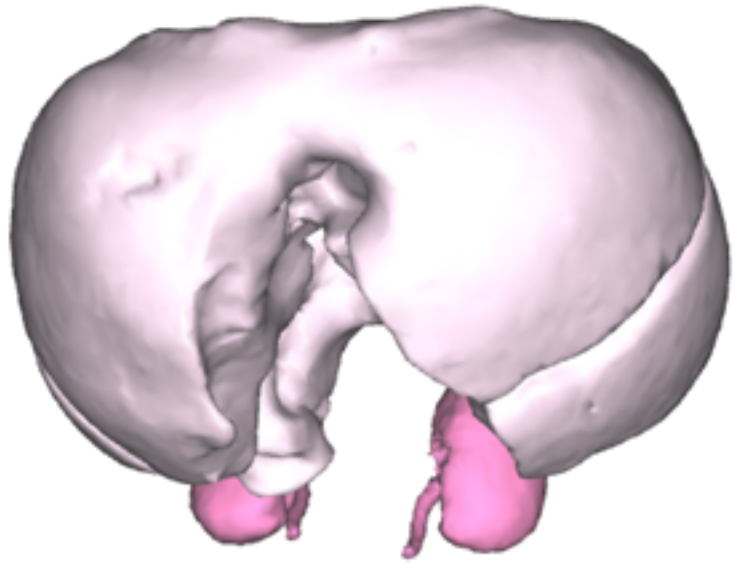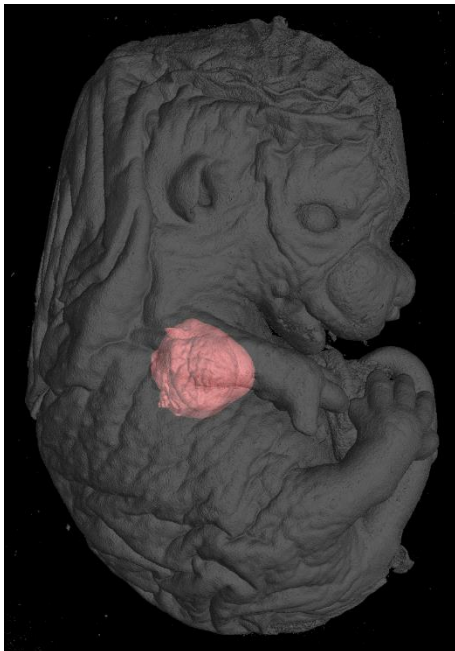

HEART

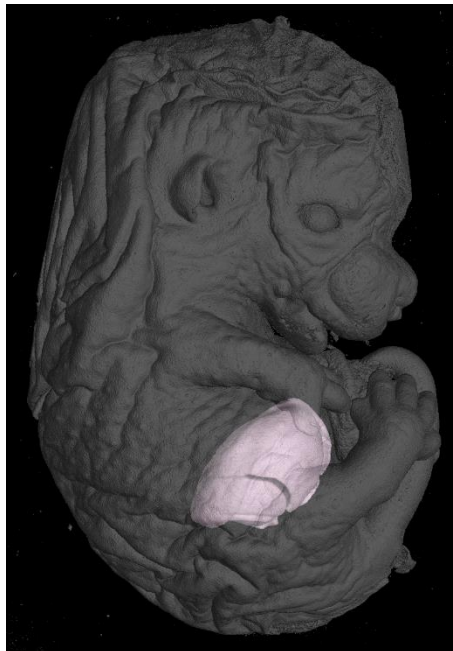

LIVER

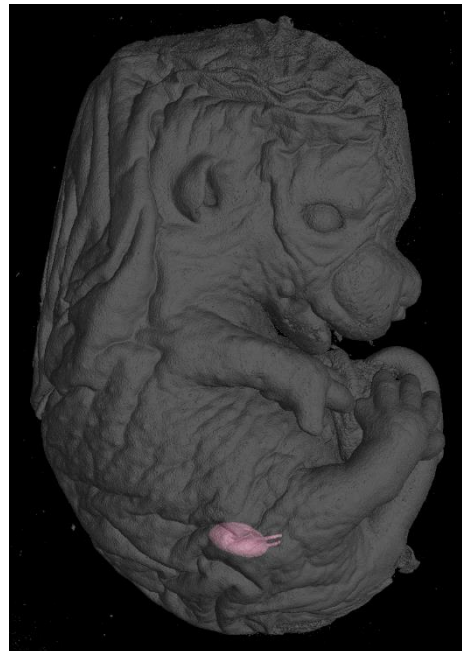

KIDNEYS

Supplement: FIGURE S3 — The 3D reconstruction of selected organs in micro-CT of control Cdk13+/+ and Cdk13tm1a/tm1a embryos. Overall view on embryos of Cdk13+/+ (A) and Cdk13tm1a/tm1a (B) with 3D reconstruction of heart, liver and kidney (left). Gross morphology is well visible on movable model on the right side. To visualize only one organ, click on the lower row and select heart (orange), liver (pink) or kidney (purple) organ only. Scale bar = 1 mm. [file Image_3.pdf]
